# The Application of Machine Learning Algorithms to Predict HIV Testing Using Evidence from the 2002–2017 South African Adult Population-Based Surveys: An HIV Testing Predictive Model †

**DOI:** 10.3390/tropicalmed10060167

**Published:** 2025-06-14

**Authors:** Musa Jaiteh, Edith Phalane, Yegnanew A. Shiferaw, Haruna Jallow, Refilwe Nancy Phaswana-Mafuya

**Affiliations:** 1 South African Medical Research Council/University of Johannesburg Pan African Centre for Epidemics Research Extramural Unit, Faculty of Health Sciences, University of Johannesburg, Johannesburg 2006, South Africa; edithp@uj.ac.za (E.P.); refilwep@uj.ac.za (R.N.P.-M.); 2 Department of Statistics, Faculty of Science, University of Johannesburg, Johannesburg 2006, South Africa; yegnanews@uj.ac.za; 3 Department of Mathematics (Data Science Option), Pan African University Institute for Basic Sciences, Technology and Innovation, Juja P.O. Box 62000 00200, Kenya; hjallow86@gmail.com

**Keywords:** HIV, AIDS, HIV testing, machine learning, predictive model, predictors, support vector machine, decision tree, random forest, logistic regression, South Africa, SABSSM, HSRC

## Abstract

There is a significant portion of the South African population with unknown HIV status, which slows down epidemic control despite the progress made in HIV testing. Machine learning (ML) has been effective in identifying individuals at higher risk of HIV infection, for whom testing is strongly recommended. However, there are insufficient predictive models to inform targeted HIV testing interventions in South Africa. By harnessing the power of supervised ML (SML) algorithms, this study aimed to identify the most consistent predictors of HIV testing in repeated adult population-based surveys in South Africa. The study employed four SML algorithms, namely, decision trees, random forest, support vector machines (SVM), and logistic regression, across the five cross-sectional cycles of the South African National HIV Prevalence, Incidence, and Behavior and Communication Survey (SABSSM) datasets. The Human Science Research Council (HSRC) conducted the SABSSM surveys and made the datasets available for this study. Each dataset was split into 80% training and 20% testing sets with a 5-fold cross-validation technique. The random forest outperformed the other models across all five datasets with the highest accuracy (80.98%), precision (81.51%), F_1_-score (80.30%), area under the curve (AUC) (88.31%), and cross-validation average (79.10%) in the 2002 data. Random forest achieved the highest classification performance across all the dates, especially in the 2017 survey. SVM had a high recall (89.12% in 2005, 86.28% in 2008) but lower precision, leading to a suboptimal F_1_-score in the initial analysis. We applied a soft margin to the SVM to improve its classification robustness and generalization, but the accuracy and precision were still low in most surveys, increasing the chances of misclassifying individuals who tested for HIV. Logistic regression performed well in terms of accuracy = 72.75, precision = 73.64, and AUC = 81.41 in 2002, and the F_1_-score = 73.83 in 2017, but its performance was somewhat lower than that of the random forest. Decision trees demonstrated moderate accuracy (73.80% in 2002) but were prone to overfitting. The topmost consistent predictors of HIV testing are knowledge of HIV testing sites, being a female, being a younger adult, having high socioeconomic status, and being well-informed about HIV through digital platforms. Random forest’s ability to analyze complex datasets makes it a valuable tool for informing data-driven policy initiatives, such as raising awareness, engaging the media, improving employment outcomes, enhancing accessibility, and targeting high-risk individuals. By addressing the identified gaps in the existing healthcare framework, South Africa can enhance the efficacy of HIV testing and progress towards achieving the UNAIDS 2030 goal of eradicating AIDS.

## 1. Introduction

South Africa remains the epicenter of HIV/AIDS, presenting a significant public health challenge despite years of intervention efforts. Currently, about 8 million individuals are living with HIV/AIDS (PLWHA), representing the highest number of HIV cases globally, with 17.1% of all PLWHA being adults [1,2]. The country has implemented various strategic initiatives to enhance HIV prevention and treatment programs aimed at curbing the virus’s spread. These efforts have led to noteworthy progress, with South Africa having the largest number of people on antiretroviral treatment (ART) [1,2]. However, the South African National Strategic Plan (NSP) for HIV, tuberculosis, and sexually transmitted infections (2023–2028) indicates a continued need to promote the uptake of HIV testing services to ensure that all PLWHA are identified and receive appropriate treatment [2].

HIV testing is an essential intervention within the HIV care continuum and remains the only means to diagnose and link PLWHA to care [3]. From the early to mid-2000s, HIV testing rates have steadily increased globally, particularly in regions severely affected by the epidemic [4,5,6]. The South African National HIV Prevalence, Incidence, Behavior, and Communication Survey (SABSSM) has been tracking the country’s HIV epidemic since 2002 [7]. From the first survey in 2002, the prevalence of HIV testing has significantly risen from 21.4% to 75.2% in 2017, with 90% of PLWHA aware of their HIV status by the close of 2022 [7,8,9,10,11,12]. Although this progress is commendable, it still falls short of the 2023–2028 NSP and Joint United Nations Program on HIV/AIDS (UNAIDS) 95-95-95 goal [2,13]. Several barriers contribute to this shortfall, including stigma, discrimination, limited access to testing among key populations (KPs), and socioeconomic and behavioral factors [5,6,13,14,15,16]. Moreover, provincial reports from a recent SABSSM survey (SABSSM VI) indicate advancements in ART uptake but highlight numerous challenges related to HIV testing [14]. Testing challenges still persist despite the efforts to roll out universal screening services [7,14].

Conventional statistical methods have been widely used in identifying pertinent factors associated with HIV testing in South Africa. Evidence from a series of SABSSM surveys by Jooste et al. [6] reveals that being young, male, unmarried, unemployed, and residing in rural areas are major barriers to HIV testing. HIV testing coverage is still low in areas like uMkhanyakude, Udu, KwaZulu-Natal, and Vhembe in Limpopo compared to urban areas [15]. In addition, HIV testing remains significantly low among key populations and older adults experiencing HIV-related stigma in rural South Africa [5,16]. Given the ongoing challenges with HIV, coupled with post-COVID-19 issues, it is imperative to apply innovative methods to explore new factors that could enhance HIV testing. While traditional statistical models are beneficial, they frequently fail to accurately forecast HIV risk when applied to complex datasets [17]. This is due to their limited ability to effectively analyze complex data, weak predictive accuracy, and inflexibility [18]. On the other hand, machine learning (ML) is found to be a practical statistical tool for predicting HIV risk/testing using complex datasets, hence potentially improving HIV prevention [18,19].

ML is a branch of artificial intelligence (AI) that involves the use of algorithms enabling machines to learn from data and make predictions or decisions without being explicitly programmed [20]. There are various types of ML, including supervised ML (SML), unsupervised ML (UML), and reinforcement learning [21]. In SML, a model learns from data patterns by training it with labeled data, where each input has a corresponding output [22]. Examples of SML include support vector machines (SVM), random forest, decision trees, logistic regression, extreme gradient boosting (XGBoost), deep learning models, etc. [21]. On the contrary, UML uncovers data patterns or relationships without labelling the data [22]. Reinforcement learning is also a common ML where prediction is influenced by the external environment [21]. The use of ML has become popular in recent years, especially in the realm of healthcare, due to its ability to learn from data to improve the efficiency and accuracy of predictions or decisions [23]. ML models increase early prediction of HIV transmission, enable viable testing methodologies to improve testing service efficiency, and optimize resource allocation, all of which contribute to enhanced HIV testing [18].

Recent studies have demonstrated that ML methods provide better accuracy in predicting HIV testing compared to traditional methods [17,18,20,24,25,26,27]. He et al. [24] compared ML approaches with traditional statistical methods and found that random forest was more accurate than traditional logistic regressions in predicting HIV infection among men who have sex with men (MSM) in China. A penalized logistic regression, a generalized additive model, SVM, and gradient boosting trees were used to analyze Demographic and Health Survey data from 10 eastern and southern African countries [28]. The findings show that gradient-boosting trees and SVM exhibit high F_1_-scores, predicting the HIV status of males (76.8%) and females (78.8%), respectively. The risk of HIV was associated with age, place of residence, condom use, number of partners, and wealth index [28]. An advanced ML technique obtained reliable results in estimating HIV/AIDS prevalence across the nine provinces in South Africa [29]. With an accuracy of 81.29%, the decision tree identified age, knowledge of HIV testing, sex debut, sexual activity, and contraceptive use as the main predictors of HIV testing among adolescents in Ethiopia. In a study by Ji et al. [30], the random forest achieved an accuracy of 99% in developing an HIV/STI risk prediction model. This automatic classification model is convenient and enhances cost-effective HIV testing [30].

ML offers several benefits over traditional statistical methods in developing predictive models aimed at strengthening HIV testing services [17,18,19]. Its high accuracy and efficiency in handling complex data give it a distinct edge, making it a valuable tool for predicting public health decisions, especially in South Africa, where stigma and sociobehavioral and economic factors continue to hamper testing efforts [16,18]. As a matter of fact, the recent cutback of funding for HIV interventions is concerning and highlights the urgent need for practical ML applications to optimize policy health decisions under resource constraints [31]. However, empirical evidence shows limited application of ML in informing testing policies in South Africa [18,32]. This highlights the importance of investing in training programs, capacity development, infrastructure upgrades, and stakeholder engagement to fully realize ML’s potential. Leveraging ML will not only improve targeted testing but also support South Africa in achieving the 95-95-95 goal of ending the HIV epidemic.

Despite the availability of research data on the factors associated with HIV testing, in South Africa, no study has been found to apply ML techniques across the five cycles of the SASSM data. Hence, this study applied four SML algorithms to determine the consistent predictors of HIV testing among South African adults using the five cycles of the SABSSM survey. The findings of this study will be used to develop an evidence-based framework to enhance HIV testing in South Africa. The framework will be guided by the best ML model from the analysis to inform and strengthen HIV testing policies and programs for South African adults.

## 2. Materials and Methods

### 2.1. Study Design

This retrospective, cross-sectional analysis utilized datasets from repeated population surveys in South Africa. We analyzed secondary datasets (SABSSM 2002, 2005, 2012, and 2017), which were originally conducted by the Human Science Research Council (HSRC) using cross-sectional designs with stratified multi-stage sampling techniques [7,8,9,10,11,12,33]. The study employed SML algorithms to identify factors associated with HIV testing among South African adults aged 18 and older, using data from five different SABSSM surveys. Four SML algorithms, namely, logistic regression, SMV, random forest, and decision trees, were used in developing an HIV testing predictive model. The four ML models developed an effective predictive model for identifying and strengthening HIV testing in South Africa. A detailed research protocol was developed to design this study’s methodology and published elsewhere [33].

### 2.2. Study Setting

**Figure 1:**
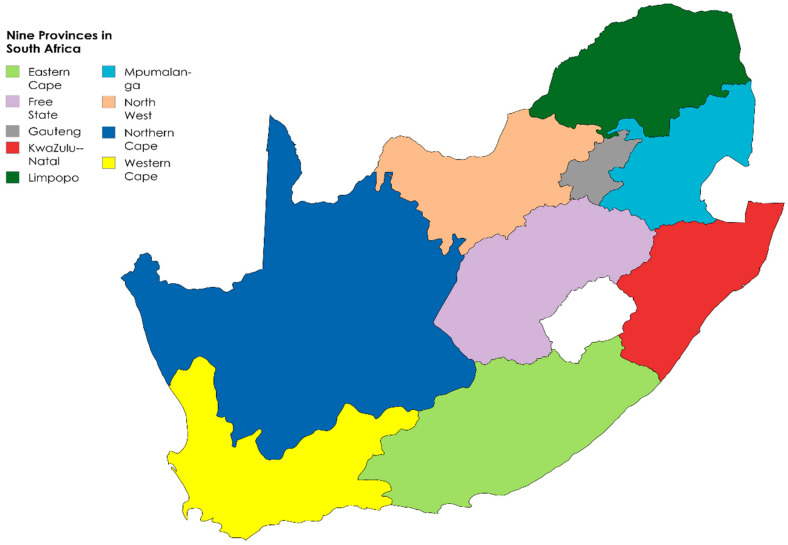
The map of South Africa (created using mapchart.net).

This study analyzed nationwide survey datasets—five cycles of the SABSSM survey. The surveys were conducted across the nine provinces of South Africa, including the Western Cape, Eastern Cape, Northern Cape, Free State, KwaZulu-Natal, North West, Gauteng, Mpumalanga, and Limpopo (see Figure 1 for geographical location). South Africa, a country with a population of over 63 million, has about 8 million people living with HIV [1,34]. This is the highest number of HIV cases globally.

### 2.3. Data Source and Study Population

This analysis involved the five cycles of the SABSSM survey datasets. The SABSSM survey is a series of nationally representative cross-sectional surveys conducted by the HSRC at five-year intervals to monitor South Africa’s HIV epidemic [7]. Since the beginning of the survey, it was conducted in 2002, 2005, 2008, 2012, 2017, and 2022 [7,8,9,10,11,12]. Based on the datasets available from the HSRC [35], this study used the five cycles (SABSSM 2002, SABSSM 2005, SABSSM 2008, SABSSM 2012, and SABSSM 2017). The surveys included a representative sample of the South African population using multi-stage stratified random cluster sampling methods. The SABSSM datasets are publicly accessible upon request through the HSRC website. They were provided for this study with specific conditions, such as ethical approval and data usage agreements. Our study focused on adults aged 18 years and older across the nine provinces of South Africa. The HSRC team weighed some of the sample sizes, and we cleaned them prior to conducting the analysis. The sample sizes for each cleaned dataset are as follows: SABSSM 2002 (*n* = 6228), SABSSM 2005 (*n* = 14,285), SABSSM 2008 (*n* = 11,833), SABSSM 2012 (*n* = 24,263), and SABSSM 2017 (*n* = 35,071).

### 2.4. Inclusion and Exclusion Criteria

The SABSSM surveys involved individuals of all ages, including children, adolescents, and adults. This study focused on male and female participants aged 18 years and older who provided a definitive response (Yes or No) to the outcome variable (“*ever_hiv_test*”) during the survey.

This study excluded individuals who were below 18 years old and did not provide a definitive response (Yes or No) to the outcome variable (“*ever_hiv_test*”) during the time of the original survey. Participants who did not respond, missing data from the outcome variable “ever HIV test,” and those with more than 10% missing data were also excluded from the analysis.

### 2.5. Study Variables

We conducted data exploration using an abstraction tool [33] with headings such as “study year,” “sample size,” “study population,” “age groups,” “study setting,” and “variables”” to understand the features of the SABSSM survey datasets [33]. The outcome variable for this study was referred to as “ever HIV test” within all the surveys. If an adult reported having ever tested for HIV, the response was coded as “Yes,” whereas if an adult never tested for HIV, the response was coded as “No.” The explanatory variables (predictors) were the sociodemographic, sociocultural, socioeconomic, medical, sexual, and behavioral factors, as well as knowledge and perception in relation to HIV. The list of all the selected variables can be found in our published study protocol [33] and Table S1. Significant variables (features) were selected in different phases, as detailed in the section below.

### 2.6. Data Preprocessing and Feature Selection

#### 2.6.1. Data Exploration

Five SABSSM datasets were explored to understand the data structure and nature of the variables in each dataset to develop an HIV testing predictive model. The outcome variable (*ever_hiv_test*) refers to individuals who reported having ever tested for HIV, and it was used as a benchmark in finalizing the sample for each SABSSM dataset. Due to the large number of missing data, only those who provided a definitive answer (i.e., Yes or No) to the outcome variable (*ever_hiv_test*) were included in the analysis. Individuals within all the “missing” and “No response” categories were excluded from the data. This was to ensure clarity and accuracy in the outcome classification. This procedure lowered the sample sizes compared to the original datasets. Potential predictors were first identified through a comprehensive literature review on the variables influencing HIV testing uptake. We only included variables with missing data rates of <10% during the data cleaning (Table S1). We encountered many missing values in the dataset, which affected the selection of our choice variable to a certain extent.

#### 2.6.2. Data Imputation

Variables that met the <10% missing threshold were all recorded in categories, and a simple imputation method (mode) was used to treat missing values. This imputation method is used due to its simplicity and effectiveness for categorical variables [36]. In addition, we applied mode imputation to ensure that the distribution of the categories of the original data was preserved, given the high dimensionality of the dataset. Although this method could introduce bias, especially if the most frequent value is overrepresented, we included variables with a <10% missing rate.

#### 2.6.3. Feature Selection

**Figure 2:**
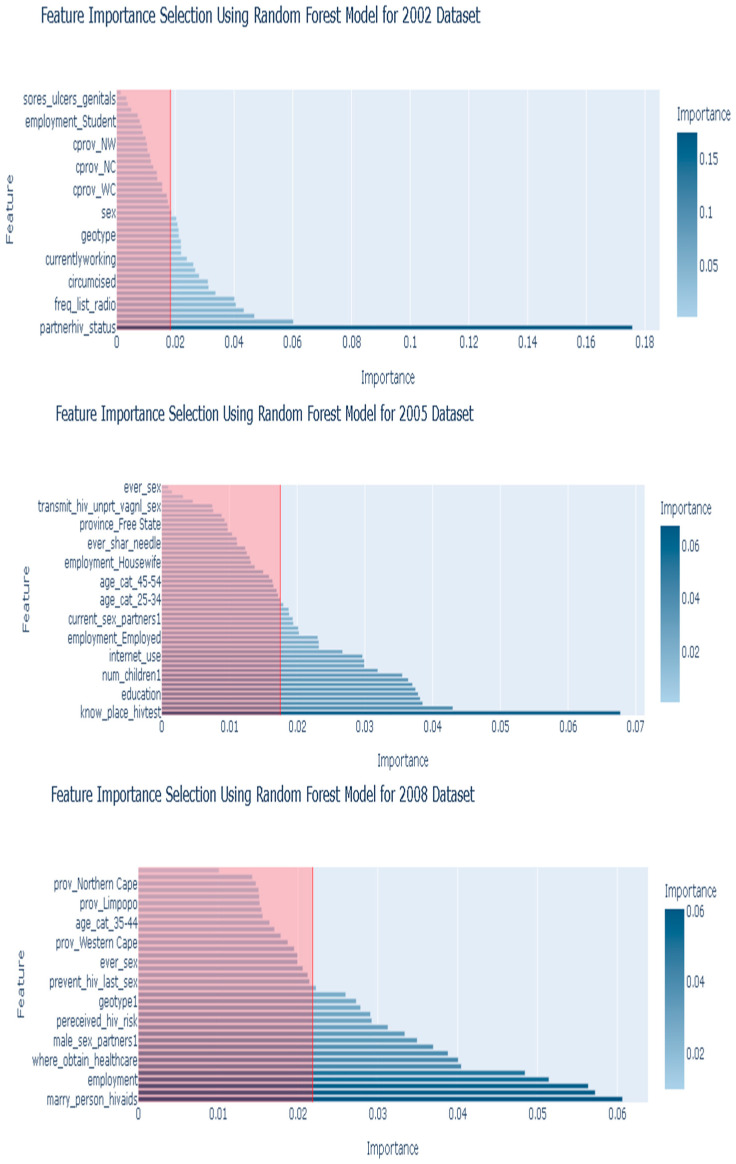

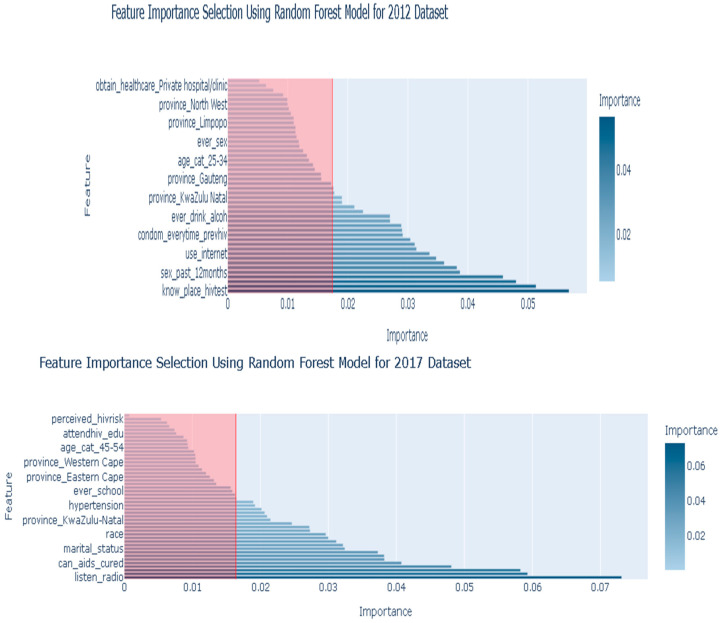
Feature importance selection using the random forest model. Note: The minimum threshold for feature importance was set at 50th percentile; red area shows variables below the threshold.

To select the important predictors (features), we first applied one-hot encoding to encode the selected variables. Since all the variables were categorical, this procedure transformed each category into binary variables, represented by 0 and 1. The encoding ensures the applicability of ML algorithms to the variables [37]. Thereafter, a correlation analysis assessed multicollinearity between the explanatory variables before the feature selection. A correlation coefficient threshold of >0.8 indicated multicollinearity, and a few were detected in the 2002 and 2008 datasets (Figure S1). The feature selection process consisted of two phases. In the first phase, we conducted a Chi-square test to identify significant explanatory variables (predictors) associated with HIV testing. This test served as a prescreening tool for a large number of explanatory variables in the SABSSM datasets. Variables with a *p*-value of less than 0.05 were retained as inputs for the ML algorithms, which were then used to train and validate the models. The selected variables in the initial feature selection are presented in Table S1. In the second phase, the best model—random forest—was employed to further refine the feature set. By analyzing feature importance scores, the random forest identified the 20 most essential predictors from each dataset. These features are illustrated in Figure 2. This two-phase approach guaranteed that only the most relevant variables were incorporated into the final models.

### 2.7. Supervised Machine Learning Algorithms

The study utilized four SML algorithms to predict HIV testing in repeated adult population-based surveys in South Africa. Below is an overview and the mathematical formulations for the key classification models used in this study: logistic regression, SVM, random forest, and decision trees [38]. These algorithms are commonly used in developing predictive models for HIV risk/testing [18].

#### 2.7.1. Decision Tree

A decision tree makes predictions by recursively splitting the dataset based on feature conditions to minimize impurity. Entropy measures the uncertainty (or impurity) of a dataset, and information gain is used to select the feature that best reduces this uncertainty when splitting the dataset. The entropy of a dataset *S* can be given as

$$H\left(S\right)=-\sum p_{i}\log_{2}p_{i}$$

where *p_i_* is the probability of class *i.*

The Gini impurity quantifies the likelihood that a randomly selected element from the dataset will be misclassified if labeled according to the class distribution. The Gini impurity of a dataset S is defined as follows:

$$H\left(S\right)=-\sum p_{i}^{2}$$

where *p_i_* is the probability of class *i.*

Information gain evaluates the effectiveness of a feature when splitting the dataset, and is defined as follows:

$$IG\left(S,\;A\right)=H\left(S\right)-\sum \frac{\left|S_{v}\right|}{S}\;H(S_{v})\;$$

where *S_v_* represents the subsets created by splitting on attribute *A.*

#### 2.7.2. Support Vector Machine

In the case of SVMs, they function as non-probabilistic binary linear classifiers, depending on their settings [39]. They perform well on unseen data and can be extended to perform regression tasks [40]. Overall, SVMs are logical, theoretically sound, and proven effective in practice [40]. SVM is designed to find a hyperplane that maximizes the margin between two classes.

The hyperplane equation is defined as follows:

$$f\left(x\right)=w^{t}\;x+b$$

where *w* is the weight vector, *x* is the feature vector, and *b* is the bias term. The SVM objective is to maximize the margin between the two classes:

$$\underset{w,b}{min}\frac{1}{2}||w|{|}^{2}$$

subject to the constraint:

$$y_{i}\left(w^{T}x_{i}+b\right)\geq 1,\;\;\forall i$$

where *yi* ∈ {−1, 1} represents class labels.

For non-linearly separable data, the kernel trick maps the data to a higher-dimensional space:

$$K(x_{i},\;x_{j})=\emptyset (x_{i}{)}^{T}\emptyset (x_{j})$$

where *ϕ*(*x*) is the mapping function.

#### 2.7.3. Random Forest

Random forests are used to solve both regression and classification problems [33,40,41]. They are very fast to train and predict, relying on one or two tuning parameters [40]. They exhibit additional features such as measures of variable importance, differential class weighing, missing value imputation, and visualization, making them interesting statistical algorithms [40]. Random forest is an ensemble learning technique that increases predictive accuracy by aggregating the results of several decision trees. In classification tasks, it employs a voting mechanism to identify the most frequently occurring class, while in regression tasks, it averages the predictions from all trees. This methodology not only improves accuracy but also helps mitigate the risk of overfitting. The final prediction is determined by selecting the class that receives the highest number of votes from the individual trees, as follows:

$$\hat{y}=arg\;max\;\sum_{t=1}^{T}I\left(h_{t}(x\right)=c)$$

where *h_t_*(*x*) is the prediction of tree *t*, and *c* is the class.

#### 2.7.4. Logistic Regression

Logistic regression is a statistical method utilized to address classification problems by estimating the probability that a given input corresponds to a particular class. This technique is commonly applied in both binary and multinomial classification contexts, enabling the analysis of complex relationships between predictor variables and categorical outcomes [38,42]. In a specific technique, logistic regression typically states where the boundary between the classes exists, as well as how the class probabilities rely on distance from the boundary [38]. When the dataset is larger, this advances faster towards the extremes (0 and 1) [38].

This method is employed to estimate the probability that a given observation belongs to a particular class. It utilizes the sigmoid function to transform the linear output into a value that represents a probability, thus facilitating interpretation and decision-making in classification tasks. The logistic function is given by the following equation:

$$P(Y=1|X)=\frac{1}{1=e^{-(w^{T}x+b)}}$$

The binary cross-entropy is the cost function used to evaluate the model’s error:

$$j\left(w,b\right)=-\frac{1}{m}\;\sum_{i=1}^{m}[y_{i}log(\hat{y})log(1-\hat{y})]$$

where *m* is the number of samples, *y_i_* is the actual label, and $\hat{y}$ is the predicted probability.

Gradient descent is used to update the weights (*w*) and bias (*b*) in the model:

$$w:=w-\propto \frac{\partial J}{\partial w}\;,\;\;b≔b-\propto \frac{\partial J}{\partial b}$$

where *α* is the learning rate.

### 2.8. Alternative Machine Learning Models

Besides the four SML algorithms used in this study, there are various alternatives that could have been applied in similar studies. Jaiteh et al. [18] categorized the most frequently used ML models in predicting HIV risk/testing. Their results showed that XGBoost, the least absolute shrinking and selection operator, and deep learning models such as artificial neural networks, recurrent neural networks, convolutional neural networks, and long-short term memory networks were commonly used [18].

UML, which performs predictions without explicitly labelling data, in contrast to SML, could serve as a great alternative for this study [18,21]. Other methods, such as ensemble learning (combines predictions from multiple models) and reinforcement learning (output is generated by interacting with the external environment) would be useful alternatives as well [21].

### 2.9. Training and Model Validation

After selecting the significant predictors using the Chi-square test (*p* < 0.05), each dataset was split into 80% for training and validation and 20% for testing. The training and validation were conducted using a probability threshold to predict factors associated with HIV testing using the four SML algorithms (decision tree, SVM, random forest, and logistic regression). A 5-fold cross-validation technique was used, where the training sample was divided into five folds. 

### 2.10. Model Evaluation and Algorithm Selection for Predicting HIV Testing

The goal was to determine which ML, after feature selection, was most suitable for predicting HIV testing in repeated adult population-based surveys in South Africa. To evaluate this, the following metrics were considered for each model: accuracy, precision, recall, F_1_-score, area under the curve–receiver operating characteristics (AUC-ROC), and confusion matrix. Additionally, cross-validation averages were used to assess model consistency.

The study evaluates the performance of the SML models on five SABSSM datasets to predict HIV testing among South African adults. The models used include decision trees, SVM, random forest, and logistic regression.

The random forest model demonstrated the highest overall performance in the preliminary analysis, particularly on the 2002 datasets, with an accuracy of 83.80%, precision of 83.28%, recall of 83.75%, and F_1_-score of 83.51% (Table S2; Figure S2). The AUC of 91.55% is the highest across all datasets, demonstrating excellent ability to discriminate between the classes. Additionally, the cross-validation average for the random forest on the 2002 dataset is 83.01%, reflecting consistent and reliable model performance across folds. The random forest also recorded the highest accuracy in classification, with the highest number of true positives and true negatives compared to the other ML models (Figure S2). This model demonstrates the best combination of predictive power and consistency across all the datasets. However, SVM outperformed all other models in the 2002 dataset in terms of precision (90.13%), while logistic regression achieved the highest recall (84.2%) in the 2012 dataset. Overall, random forest emerged as the best model in predicting HIV testing in the preliminary analysis of the five SABSSM survey datasets. The SVM and logistic regression performed reasonably well compared to the decision tree.

The models were evaluated for both overfitting and underfitting across subpopulations, including age categories, sex, race, province, education, and employment. This was achieved through stratified performance evaluations, and each model was evaluated with metrics such as accuracy, precision, AUC, recall, and F_1_-score in each subgroup using 5-fold cross-validation. This ensured the generalizability and consistent performance of the ML models and minimized the chances of overfitting or underfitting. The selection of features with random forest using Gini impurity and mean threshold also helped to reduce overfitting.

### 2.11. Final Feature Selection and HIV Testing Predictive Modeling

The feature selection process involved identifying the most important variables using the random forest model on each dataset. Since the random forest was found to be the best model for predicting HIV across all the SABSSM datasets in the initial results, it was used to select the 20 best features for the final analysis (Figure 2).

Specifically, feature importance scores were computed based on the Gini impurity criterion, and the threshold was set at the 50th percentile, ensuring that features with at least median importance were retained. The top 20 features from each dataset were selected for training and testing all the ML models. This approach ensured that only the most relevant predictors were considered, thereby improving model interpretability and potentially reducing overfitting compared to using all the features. To evaluate the trade-off between model complexity and performance, we compared the predictive performance of models trained on all available features with those trained only on the top 50% most important features (based on random forest feature importance). The results indicated no significant drop in F_1_-score and a slight improvement in generalization, supporting the feasibility of dimensionality reduction for interpretability without compromising performance.

The four ML models were used to train, validate, and test the selected 20 features. The dataset was split into 80% training and 20% testing sets, and a 5-fold cross-validation technique was applied during the development of the final models. The selected features were consistently used for training and testing using decision trees, SVM, random forest, and logistic regression models. Furthermore, the models were evaluated using the same evaluation metrics (accuracy, precision, recall, F_1_-score, AUC, confusion matrix, and cross-validation averages).

### 2.12. Feature Importance Scores Using Random Forest

The feature importance scores from the random forest model were set at a threshold of the 50th percentile to indicate how much each feature contributes to predicting HIV testing (the target variable: *everhiv_test*). However, these scores only reflect the relative contribution of the features to making accurate predictions; they do not provide information on whether a feature has a positive or negative impact on the likelihood of HIV testing. The importance of scores were as follows:

i. Higher importance score: The feature has a stronger influence on predicting HIV testing.
ii. Lower importance score: The feature has a weaker influence on predicting HIV testing.

For example, *partnerhiv_status* (0.1757) is the most influential feature, meaning it plays the biggest role in distinguishing between individuals who have tested for HIV and those who have not. The feature *education* (0.0602) is an important factor, but contributes less than *partnerhiv_status*. Features like employment status and age category also have considerable influence.

### 2.13. Assessing Temporal Changes in Features’ Predictive Power

We analyzed changes in the predictive power of socio-behavioral factors over time, i.e., regarding the changes in the top predictors from 2002 to 2017. We conducted a temporal analysis of the feature importance rankings using a random forest classifier separately for each survey year (2002, 2005, 2008, 2012, and 2017). This approach allowed us to assess how the influence of individual predictors evolved across the 15-year period of data collection.

There was a notable change in the most important features associated with HIV testing. For instance, in 2002, the top predictors included partner’s HIV status, education, and age at first sex. By 2005 and 2008, knowledge of HIV testing locations, alcohol use, and marital status became more prominent. In the later years, particularly 2012 and 2017, the importance of media exposure (e.g., listening to radio, watching TV), internet use, and circumcision status increased significantly (Table 1).

### 2.14. Evaluating the Coefficients’ Important Features Using a Logistic Regression Model

After selecting the most important features with random forest, we used logistic regression to determine the strength and direction of the association between the outcome and independent variables. The odds ratios (OR) were reported with a significance level of *p* < 0.05 using a 95% confidence interval (95% CI). The regression model was necessary to quantify the relationship between the outcome and predictors while controlling for confounders.

**Figure 3:**
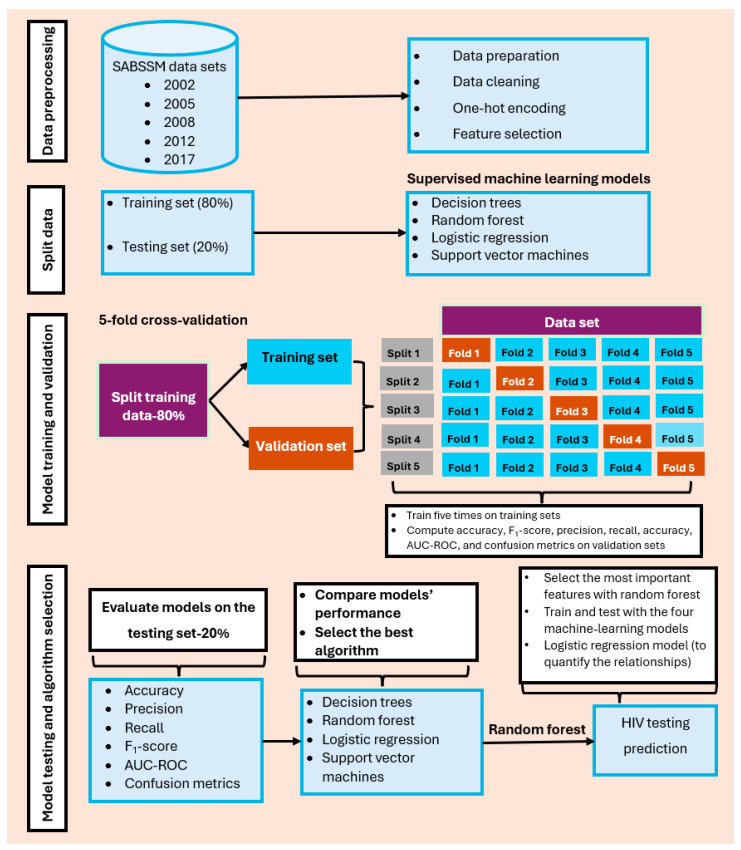
Data preprocessing and HIV testing prediction modeling (adapted from Jaiteh et al. [33]). Note: SABSSM, South African National HIV Prevalence, Incidence, Behavior and Communication Survey.

All analyses in the study were performed with Python version 3.12.0, developed by the Python Software Foundation, Wilmington, DE, USA. Figure 3 shows data preprocessing, feature selection, model training, and validation, as well as HIV testing prediction modeling using the five SABSSM datasets.

### 2.15. Risk for Bias Assessment

Several techniques were applied to mitigate biases in this study. At first, we developed a detailed study protocol [33] to guide the methodology. In addition, the Joanna Briggs Institute Critical Appraisal for Analytical Cross-Sectional Studies was used to guide criteria, variable selection, and data analysis. A data abstraction tool was used to explore and select key variables before the analysis. The datasets were thoroughly cleaned, and missing values were treated appropriately. Confounders and redundant variables were removed from the dataset. The random forest model was used to select the best feature for the final analysis.

All the datasets exhibited class imbalances, with a smaller proportion of individuals reporting ever tested for HIV. We addressed the class imbalance using the Synthetic Minority Over-sampling Technique (SMOTE) after identifying the selected predictors and the target variable. After applying SMOTE, the class distribution was balanced, resulting in equal or near-equal representation. This balancing helped mitigate bias during model training and ensured the model was not skewed toward the majority class. The transformation was consistent across all years. This was to ensure that class imbalance was well-handled before splitting the datasets into training and testing data. The SMOTE technique generates synthetic samples for the minority class by interpolating between existing minority class samples. This helps to balance the dataset by increasing the number of minority class samples [43]. SMOTE was selected after comparing it with basic Synthetic Minority Over-sampling Technique and Edited Nearest Neighbors (SMOTEENN), Adaptive Synthetic Sampling (ADASYN), and random oversampling. While ADASYN performed comparably in some datasets, SMOTE consistently yielded better F_1_-scores and lower false positives by not only oversampling the minority class but also cleaning overlapping or noisy majority instances.

**Figure 4:**
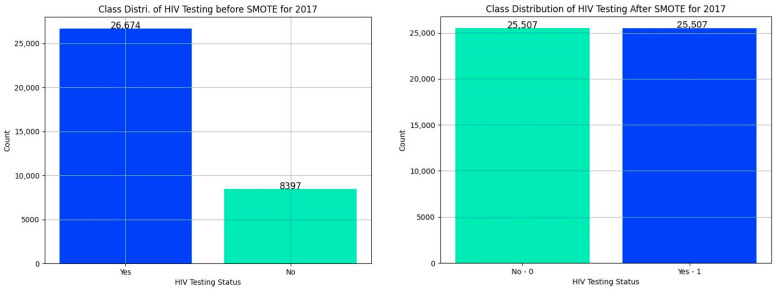
Class distribution of HIV testing before and after SMOTE for the 2017 SABSSM data. Note: SMOTE, Synthetic Minority Over-sampling Technique.

Figure 4 shows the class distribution of HIV testing before and after applying SMOTE on the 2017 SABSSM datasets. The distributions for 2002, 2005, 2008, and 2012 can be found in Figure S3.

### 2.16. Data Management

The HSRC provided the SABSSM datasets for this predictive modeling without the participants’ personal/identifying information. The researchers cleaned the datasets and stored them in a secure memory stick, only accessible to the research team. All data handling and analysis were conducted within secure, password-protected environments. The datasets were anonymized prior to analysis, and no personally identifiable information (PII) was used. The analytical workflows were documented with version control and audit trails to ensure reproducibility and compliance with data protection protocols.

The results published herein do not contain any PII of the study participants. Since this study forms a part of the Boloka project, the datasets were stored in the Boloka data repository, which is password-protected with username access. “The Boloka project aims to harness big heterogeneous data to evaluate the impact of HIV responses among key populations in generalized settings in Sub-Saharan Africa” [44]. The study complies with the Protection of Personal Information Act (POPIA) in using and managing all the available data. Importantly, the HSRC governs the utilization of the SABSSM datasets with an End-User License Agreement. According to the HSRC’s data-sharing agreements, the provided SABSSM “datasets cannot be duplicated, reshared, or sold without prior approval of the rights holder.” [35].

### 2.17. Ethical Considerations

The current study forms an integral part of a doctoral study by the first author (MJ), titled “Integration of Machine Learning Algorithms to Predict HIV Testing Associations Using Repeated Cross-sectional Survey Data in an Adult South African Population: an HIV Testing Predictive Model.” The protocol [28] was reviewed and approved by the University of Johannesburg (UJ) Research and Ethics Committee (REC), ethics approval number REC-2725-2024. Additionally, “the doctoral study falls under an umbrella project funded by the South African Medical Research Council (SAMRC) within the SAMRC/UJ Pan African Center for Epidemics Research (PACER) Extramural Unit, titled: Harnessing Big heterogeneous data to evaluate the impact of HIV responses among key populations in generalized settings in Sub-Saharan Africa” [44], ethics approval number: REC-1504-2022.

A waiver of informed consent for secondary data was given by the UJ REC before the commencement of the study. The SABSSM studies were conducted by the HSRC REC in accordance with the “International Ethical Standards and the South African Children’s Act 2007.” The datasets are protected by an End-User License Agreement, preventing their duplication, resharing, or selling without prior approval from the rights holder. The researchers adhered to all the data-sharing agreements and ethical use of secondary data.

In addition to obtaining ethics approval from the UJ REC, we observed ethical considerations regarding ML models in public health predictions, especially issues surrounding privacy, confidentiality, and potential stigma. Further safeguards were ensured by complying with the international ethics standards, such as the General Data Protection Regulation and UJ POPIA throughout the analysis. All data handling and analysis were conducted within secure, password-protected environments. The data were anonymized, and no efforts were made to re-identify the study participants.

## 3. Results

### 3.1. Prevalence of HIV Testing

The prevalence of HIV testing among individuals aged 18 years and older was reported at 26.1% (*n* = 1627) in 2002, 33.7% (*n* = 4816) in 2005, 52.6% (*n* = 6222) in 2008, 66.5% (*n* = 16,133) in 2012, and 76.1% (*n* = 26,674) in 2017 (Table 2).

The study reveals that individuals who reported having been tested for HIV in the five SABSM surveys were most likely to be Black Africans females who were aged 18–25, married, and residing in urban areas, particularly in the KwaZulu-Natal and Gauteng provinces. The prevalence of HIV testing was also high among those who attained at least a secondary education and were employed (Table 3).

### 3.2. Predictors of HIV Testing from the Five SABSSM Surveys

The main aim of this study was to identify consistent predictors of HIV testing from the SABSSM surveys. The best model (random forest) ranked these predictors according to their level of importance (Figure 2; Table 1).

In 2002, knowing a partner’s HIV status was the most important predictor of HIV testing, with an importance score of 0.1757. This was followed by education, race, age at first sexual intercourse, frequently listening to the radio, and knowing if HIV/AIDS has a cure, with each having an importance score of 0.04. Frequently watching TV, being circumcised, and drinking alcohol were also significant in predicting HIV testing.

The 2005 SABSSM indicated that knowing a place where HIV tests could be conducted was the strongest predictor of HIV testing, with a ranking of 0.677. Contraceptive methods, age at first sex, watching TV, Education status, age above 55 years, frequency of alcohol intake, race, number of children, and marital status were also among the most important predictors of HIV testing.

Furthermore, marrying a person with HIV/AIDS was the most significant predictor (0.6064) of HIV testing in the 2008 SABSSM survey. Knowing a place to where HIV testing could be conducted, highest education, employment status, race, a place where an individual obtains healthcare, knowing if AIDS has a cure, and age (55 years or older) were among the top predictors of HIV testing.

In 2012, knowing a place to take an HIV test ranked as the highest predictor of HIV testing with an important score of 0.0568. Listening to the radio, age at first sex, knowledge of male circumcision in HIV prevention, sex in the last 12 months, and watching TV also emerged as significant predictors of HIV testing.

Moreover, the 2017 data indicated that listening to the radio ranked as the top predictor of HIV testing, with an importance score of 0.0731. This was followed by knowing a place to take an HIV test, watching TV, knowledge of male circumcision in HIV prevention, knowing if AIDS can be cured, employment status, internet use, having sexual intercourse, marital status, sex, using a condom every time, alcohol intake, race, and geographical location.

The most consistent predictors of HIV testing across the five SABSSM surveys identified by the random forest model include race, condom use, alcohol intake, marital status, geographical location, and employment. Important features that predicted HIV testing in at least three SABSSM surveys are related to individuals’ education, province, sex, place where healthcare is obtained, knowledge of HIV/AIDS, knowing where to take an HIV test, listening to the radio, watching TV, internet use, perceived HIV risk, and sexual behaviors (Table S3).

To analyze changes in the predictive power of socio-behavioral factors over time, we conducted a temporal analysis of feature importance rankings for the 20 features across all the datasets with random forest (Table 1). Our findings indicate notable changes in the most important features associated with HIV testing from 2002 to 2017. In 2002, the top predictors included partner’s HIV status, education, and age at first sex. By 2005 and 2008, knowledge of HIV testing locations, alcohol use, and marital status became more prominent. In later years, particularly 2012 and 2017, the importance of media exposure (e.g., listening to radio, watching TV), internet use, and circumcision status increased significantly. These shifts may reflect broader social and behavioral changes, such as increasing access to media, improved health communication, and digital outreach programs, as well as rising educational attainment and awareness. Therefore, the top 20 features associated with HIV testing have indeed evolved over the study period, supporting the dynamic nature of the determinants of HIV testing behavior.

### 3.3. Logistic Regression Model Showing the Significant Predictors of HIV Testing

Since we selected the top 20 features as the most important predictors of HIV testing using the random forest, these are the characteristics most associated with whether an individual gets tested. However, without knowing the direction of impact, we cannot say for sure whether these individuals need more intervention or are already more likely to test. Thus, we conducted a logistic regression on the 20 features, which provides coefficients indicating positive or negative relationships. Feature importance in random forest only ranks the significance of each predictor.

We provided the results output below and analyzed the impact by comparing the distribution of features between those who tested and those who did not. We used three (2002, 2012, and 2017) datasets because they are the most suitable datasets for predicting HIV Testing, as shown by our model results across all five datasets.

The regression models show that knowing the partner’s HIV status was the strongest positive predictor (OR = 2.37), indicating that individuals with an HIV-positive partner were significantly more likely to get tested in the 2002 SABSSM data. In addition, knowing a place to take an HIV test was a consistently strong predictor (OR = 1.67 in 2012, OR = 1.62 in 2017) in both the 2012 and 2017 SABSSM surveys, respectively. Attaining higher education levels, especially secondary and above, increased the likelihood of testing for HIV in the 2002 survey. Thus, awareness of HIV testing locations increases the chances of being tested. Moreover, engaging in sexual activity in the past 12 months exhibited strong positive predictive power in the 2012 and 2017 SABSSM surveys (OR = 1.46 in 2012, OR = 1.40 in 2017). Across all years, employed individuals were more likely to go for HIV testing (OR = 1.14–1.31 across years). Likewise, people who used the internet had greater odds (OR = 1.14–1.31 across years) of undergoing an HIV test compared to those who did not.

On the contrary, the 2012 and 2017 SABSSM surveys consistently showed that males (OR = 0.72 in 2012, OR = 0.75 in 2017) and those aged 55 and older (OR = 0.68) were less likely to get tested for HIV. Circumcision was negatively associated with HIV testing (OR = 0.83 in 2002, OR = 0.96 in 2012). In addition, individuals who believed they had little or no risk of HIV were less likely to test (OR = 0.89–0.91) in the 2017 survey. Moreover, believing that AIDS can be cured consistently reduces the likelihood of HIV testing (OR = 0.90–0.95 in all years), indicating that misconceptions about HIV may discourage testing.

### 3.4. Models’ Accuracy, Precision, Recall, F_1_-Score, AUC, and Cross-Validation Averages

The study evaluates the performance of the four SML models across the five SABSSM datasets (2002, 2005, 2008, 2012, 2017) to predict HIV testing among South African adults. The models, including decision trees, SVM, random forest, and logistic regression, were evaluated using key performance metrics such as accuracy, precision, recall, F_1_-score, confusion matrix, and cross-validation averages.

**Figure 5:**
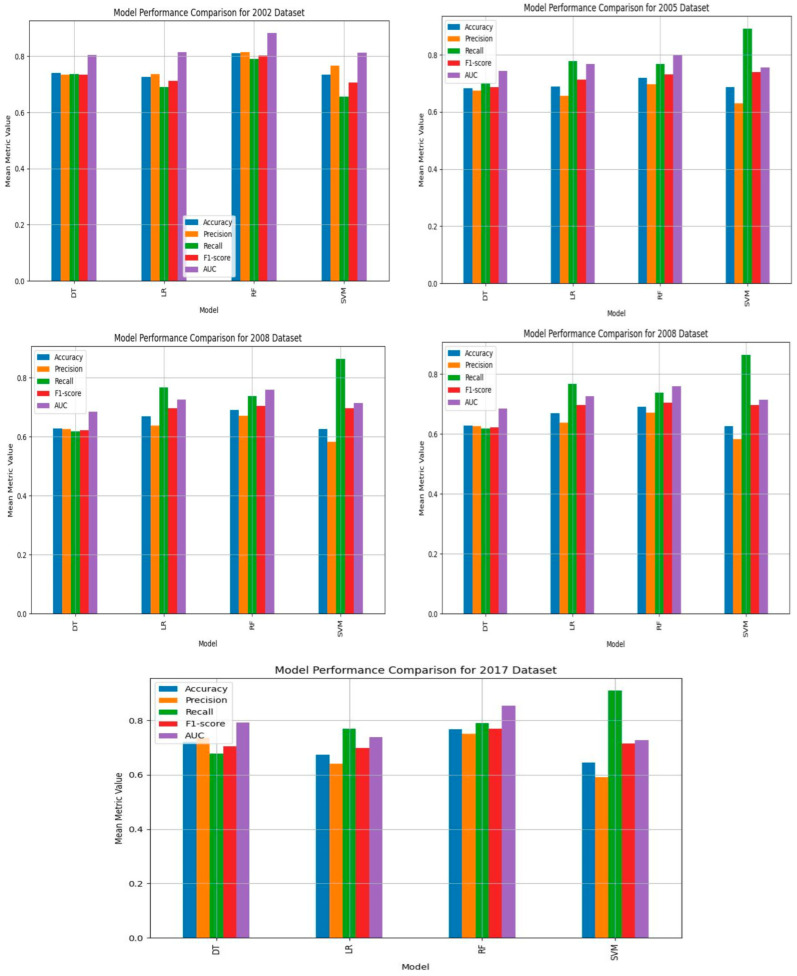
Models’ performance comparisons. DT, decision trees; SVM, support vector machines; RF, random forest; LR, logistic regression; AUC, area under the curve.

**Figure 6:**
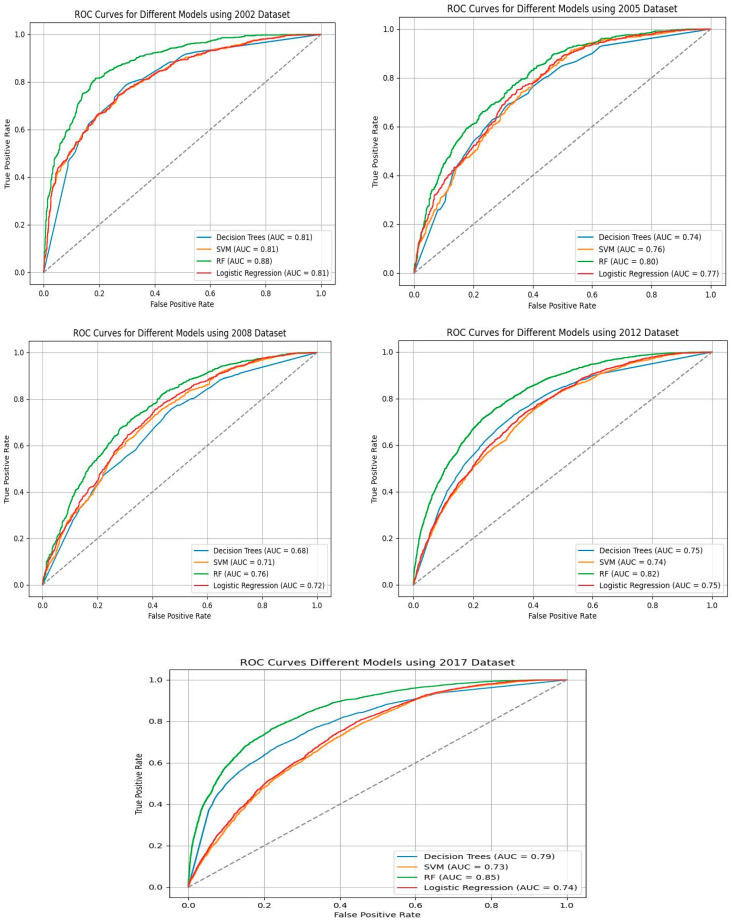
ROC curves of the different models for all five datasets. DT, decision trees; SVM, support vector machines; RF, random forest; LR, logistic regression; AUC, area under the curve. Note: The dotted diagonal line represents the performance of a random classifier (i.e., no discrimination between classes).

The 2002 dataset demonstrated the best predictive performance with all four models, with random forest being the most effective. Random forest achieved an accuracy of 80.98%, a precision of 81.51%, a recall of 79.12%, an F_1_-score of 80.30%, and an AUC of 88.31. The cross-validation average was 79.10%. The superior performance of random forest on this dataset suggests that the 2002 data contain the most informative features for predicting HIV testing. The high AUC value (88.31%) indicates strong discriminatory capability between individuals who have and who have not undergone HIV testing (Table 4; Figure 5 and Figure 6).

The 2017 and 2012 datasets also demonstrated strong predictive power, particularly in recall and F_1_-score, indicating that models trained on these datasets effectively capture patterns related to HIV testing behavior. The random forest on the 2017 dataset achieved an accuracy of 76.79%, a precision of 75.17%, a recall of 78.99%, an F_1_-score of 74.81%, and an AUC of 74.82%, with a cross-validation average of 73.61%. These results indicate that both datasets have valuable predictors, though their predictive power is lower than that of the 2002 dataset. The consistency of the random forest’s performance in these datasets suggests that the feature selection was effective in enhancing model interpretability and reducing overfitting (Table 4; Figure 5 and Figure 6).

The 2005 and 2008 datasets yielded lower performances across all the ML models. The random forest model remained the top performer, but its metrics were weaker compared to the 2002, 2017, and 2012 datasets. On the 2005 data, the random forest model achieved an accuracy of 71.81%, a precision of 69.60%, a recall of 76.8%, an F_1_-score of 73.03%, and an AUC of 79.90%, with a cross-validation average of 72.60%. Similarly, the random forest model on the 2008 dataset achieved an accuracy of 69.07%, a precision of 67.14%, a recall of 73.80%, an F_1_-score of 70.31%, and an AUC of 75.94%, with a cross-validation average of 69.80%. These results suggest that while the models can still provide useful insights, the 2005 and 2008 datasets may lack some key predictive variables found in the later datasets. The relatively lower AUC values indicate that these datasets do not discriminate between tested and untested individuals (Table 3; Figure 3 and Figure 4).

Across all the datasets, random forest consistently outperformed other models, followed by logistic regression and decision trees. SVM exhibited moderate performance, excelling in recall but showing lower accuracy. Overall, random forest performed the best, with the highest accuracy and AUC across all the datasets. Logistic regression performed well, but was slightly lower than in the random forest. Decision trees demonstrated moderate accuracy but were prone to overfitting. Initially, SVM had a high recall but lower precision, leading to a suboptimal F_1_-score. To improve classification robustness, we implemented a soft-margin SVM by introducing the regularization parameter C. A value of C = 0.1 improved the F_1_-score across datasets, suggesting that allowing for a small number of misclassifications led to better generalization, particularly in the presence of noise.

### 3.5. Confusion Matrices of the Machine Learning Models

The analysis of the confusion matrices across the different datasets reveals that the random forest model consistently outperformed all the other models in terms of accuracy, precision, and recall. Across all five datasets (2002, 2005, 2008, 2012, and 2017), random forest still achieves the highest number of true positives and negatives while maintaining a strong balance between precision and recall. The SVM, despite excelling in recall, suffers from a high number of false positives, reducing its overall precision. Decision trees perform worst, with the highest number of misclassifications, while logistic regression shows a moderate performance but fails to surpass random forest in predictive capability. Notably, random forest effectively handles the increasing data compressibility in later years (i.e, 2012 and 2017), demonstrating its robustness in classification tasks (see Table 5). The 2017 dataset exhibits the best performance for the random forests, with the highest classification accuracy of 3972 true positives and 3863 true negatives. While SVM’s high recall indicates its strength in capturing positive cases across the datasets, it was poor in precision due to excessive false positives, making it less reliable. The overall findings confirm that random forest is the most effective model for this classification problem, delivering superior accuracy and balanced classification performance across all the datasets.

## 4. Discussion

### 4.1. Key Findings

This study applied four SML algorithms to identify factors associated with HIV testing among South African adults using data from five cycles of the SABSSM surveys (2002, 2005, 2008, 2012, and 2017). The performances of the ML models, namely, decision trees, random forest, SVM, and logistic regression, were evaluated using metrics such as accuracy, precision, recall, F_1_-score, AUC, confusion matrix, and cross-validation averages. The random forest model emerged as the best model in the preliminary results, and it was used to select the 20 most important features used for the HIV testing prediction modeling.

The findings revealed an increased uptake of HIV testing among South African adults from 2002 to 2017, more so among females. Our findings are consistent with published SABSSM survey reports highlighting an increase in HIV testing [7,8,9,10,11,12].

The study shows that knowing a partner’s HIV status, the place where to take an HIV test, an individual’s level of education, receiving HIV education through the media (radio, TV, internet), sexual behaviors, being female, and being a younger adult were the most important consistent predictors of HIV testing.

Random forest achieved the highest accuracy after the 20 features from each dataset were trained and tested on the four ML models. Our results are consistent with various studies where random forest outperformed other ML models [24,45,46]. Logistic regression, SVM, and decision trees performed moderately well across the datasets. Among all the datasets, the 2002 dataset achieved the best predictive performance, followed by the 2017 and 2012 SABSSM survey data.

### 4.2. Predictors of HIV Testing

We used random forest to rank the 20 most significant features, as it emerged as the best model in the preliminary analysis. The threshold for selection was set at the 50th percentile using the Gini impurity criterion. The random forest model selected the important predictors but could not demonstrate whether they were positively or negatively associated with HIV testing. Hence, we used a multivariable logistic regression model to understand the direction of the impact of the selected predictors on HIV testing among South African adults.

Based on the random forest selection, knowing a partner’s HIV status was ranked as the most important predictor of HIV testing, with a score of 0.1757 in the 2002 data. Likewise, the logistic regression model reveals that knowing a partner’s HIV status was the strongest positive predictor (OR = 2.37), indicating that individuals with an HIV-positive partner are significantly more likely to get tested in the 2002 SABSSM data. Individuals with known HIV partners are prioritized through index testing programs due to their high exposure. A study in Tanzania used different ML models to predict 86% of female partners of HIV-positive individuals [45]. Likewise, a case-finding/index testing intervention screened 51.2% of all the contacts of HIV cases from health facilities within 12 districts in South Africa [47].

Education, with an importance score of 0.6018, strongly predicted HIV testing in 2002. In the same data, the regression model asserted that obtaining higher education levels, especially secondary and above, increased the likelihood of testing for HIV. Thus, awareness of HIV testing locations increases the chances of being tested. Our results are consistent with those of Mutai et al. [48], who developed ML models from population-based impact assessment data from four Sub-Saharan African (SSA) countries. The findings revealed that an individual’s highest level of education was among the top eight predictors of targeted HIV screening [48].

Frequently listening to the radio exhibited higher scores compared to watching TV or using the internet in 2002. Listening to the radio (0.0406) was widely common in the early 2000s, possibly giving people access to HIV information and encouraging them to get tested. On the contrary, internet use was relatively scarce, which explains its lower predictive score (0.0220) in the 2002 data but higher predictive score in later years (0.0382 in 2017). Our temporal analysis also indicated changes in the predictive power of HIV testing predictors from 2002 to 2017. While partner’s HIV status, education, age at first sex, knowledge of HIV testing locations, alcohol use, and marital status significantly influenced HIV testing in 2002, 2005, and 2008, media exposure (e.g., listening to radio, watching TV), internet use, and circumcision status were more prominent in later years (2012 and 2017). In recent years, there has been a proliferation in the use of Internet/web-based services to enhance HIV testing [49]. Although Catwell et al. [49] recommend the development of standards of care for such services in Australia due to their questionable reliability, digital-based HIV testing was found to be effective in South Africa [44]. Van Heerden et al. [50] developed an AI conversational agent for mobile devices to facilitate self-HIV screening in South Africa, and the participants welcomed the innovation as a game changer. Similarly, mobile-based HIV testing was found to be more cost-effective than facility-based testing in Ethiopia [51].

Knowing a place to take an HIV test was consistently the highest predictor of HIV testing in 2005 and 2012 and the second highest in the 2008 and 2017 SABSSM datasets, with importance scores ranging from 0.06 to 0.07. Similarly, knowing a place to take an HIV test consistently exhibited greater odds of HIV testing in both the 2012 and 2017 SABSSM surveys (OR = 1.67, OR = 1.62), respectively. Supporting evidence by Petrol et al. [52] highlighted that urban American men who knew HIV testing locations were more likely to get tested than those who did not. A similar study among Ethiopian youths employed decision trees and indicated that knowing an HIV testing location was among the five top predictors of HIV testing [53].

Moreover, engaging in sexual activity in the past 12 months exhibited strong positive predictive power in the 2012 and 2017 SABSSM surveys (OR = 1.46 in 2012, OR = 1.40 in 2017). Sexual activity-related variables were also among the 20 top predictors selected by the random forest in 2012 and 2017 data. Similar results were highlighted by Alie et al. [53], whose ML-based analysis identified recent sexual activity and age at first sexual intercourse as key predictors of HIV testing.

Being employed and living in urban areas, particularly in KwaZulu-Natal, slightly increased the odds of HIV testing across multiple datasets. This indicates that socioeconomic disparities in wealth index and urban–rural settlement play critical roles in HIV testing [52,54]. According to a recent HSRC report, KwaZulu-Natal has the second-highest HIV prevalence in South Africa, yet has progressed in testing, as 94.0% of PLWHA aged 15 years and older are aware of their HIV status [55]. The high HIV prevalence in the region attracts more testing intervention efforts from the government, the Department of Health, and relevant stakeholders.

On the other hand, the 2012 and 2017 SABSSM surveys consistently showed that males (OR = 0.72 in 2012, OR = 0.75 in 2017) and those aged 55 and older (OR = 0.68) were less likely to get tested for HIV compared to females and younger adults, respectively. Our findings are consistent with a study conducted in South Africa, stating that young adults had higher odds of HIV testing, and males exhibited lower odds [56]. Various studies reveal that females are more likely to test for HIV compared to males [24,47,54,57]. This could be due to the fact that men are perceived to be at low risk of HIV compared to women [48,56,57,58], thus negatively influencing their testing behaviors. Furthermore, the study reveals that circumcision was negatively associated with HIV testing (OR = 0.83 in 2002, OR = 0.96 in 2012), which could also be associated with a perceived lower HIV risk in circumcised men. In addition, individuals who believed they had little or no risk of HIV were less likely to test (OR = 0.89–0.91) in the 2017 survey. Moreover, believing that AIDS can be cured consistently reduces the likelihood of HIV testing (OR = 0.90–0.95 in all years), indicating that misconceptions about HIV may discourage testing.

Overall, knowledge of testing centers, partner’s HIV status, and higher education strongly influenced the testing behavior of South African adults. In addition, women, employed individuals, those engaging in sexual activity, and younger adults were more likely to test. Frequent use of media, such as listening to the radio, watching TV, and using the internet, showed a slightly higher likelihood of HIV testing. The 20 traits consistently identified as important predictors across all survey years reflect well-documented social determinants of health. Among them, variables such as education level, access to media (radio TV and internet), marital status, employment, sexual behaviors, geographical location, race, sex and perceived HIV risk likely influence knowledge, awareness, and decisions around testing. Their recurring importance aligns with the existing literature on HIV prevention and behavioral models [48,49,50,51,52,53,54,55,56,57,58]. Essentially, the ranking of important predictors with random forest forms an evidence-based approach for targeting priority and high-risk individuals for HIV testing.

### 4.3. Model Performances

The study reveals that random forest is the best ML model for predicting HIV testing associations using repeated cross-sectional adult population-based surveys. Random forest outperformed all other ML models across the five datasets in terms of accuracy (80.98%), precision (81.5%), F_1_-score of 80.30%, AUC of 88.31, and cross-validation average (79.10%) on the 2002 data. The superior performance of random forest on this dataset suggests that the 2002 data contain the most informative features for predicting HIV testing. The confusion matrix shows that the 2017 dataset exhibits the best performance for the random forests, with the highest classification accuracy of 3972 true positives and 3863 true negatives. The 2005 and 2008 datasets yielded lower performances across all the ML models. The random forest remained the top performer, but its metrics were weaker compared to the 2002, 2017, and 2012 datasets.

Across all the datasets, random forest consistently outperformed other models, followed by logistic regression and decision trees. Random forest performed the best overall, with the highest accuracy, precision, F_1_-score, and AUC across all the datasets. These findings imply that the random forest model is a valuable tool for accurately classifying individuals’ HIV testing status. The model’s high F_1_-score maintains balanced precision and recall, categorizing fewer false positives and most true positives. While false negatives may increase the spread of HIV, false positives pose risks such as the discrimination and depression associated with HIV [18]. Hence, the findings show that public health interventions could rely on random forest for correctly identifying individuals who have never tested, thus optimizing resource allocation in HIV testing programs. Furthermore, the high AUC highlights the random forest’s effectiveness in predicting HIV testing across diverse populations. This is crucial for designing HIV testing in South Africa, whose population is made up of various sociodemographic groups. Our findings are in line with several other studies. A systematic review of ML algorithms indicated that random forest was an outstanding model compared to others [18]. A study in the USA [46] found that random forest was more accurate in classifying HIV testing than logistic regression. Similarly, He et al. [21] emphasized the need to consider random forest in predicting HIV risk among Chinese MSM due to its dominance in accuracy over decision trees, SVM, and logistic regression. A study in Tanzania [45] also demonstrated that random forest achieved the best results compared to other models. Random forest is a powerful ML algorithm that handles multiple regression and classification tasks, possibly making it suitable for predictions in secondary data [40,41].

SVM exhibited moderate performance, excelling in recall (89.12% in 2005, 86.28% in 2008) but showing lower accuracy and precision, leading to a suboptimal F_1_-score in the initial analysis. Since SVM is prone to misclassification in the presence of noise [59], we further applied a soft margin to the SVM, which improves its classification robustness and generalization. This adjustment improved the SVM’s metrics, yet the accuracy and precision are still low in most surveys, increasing the chances of misclassifying individuals as having been tested for HIV. Our findings are inconsistent with those of Barbieri et al. [60], who revealed that SVM achieved the highest accuracy in correctly classifying true positive and false HIV test results when compared to other ML models. In this study, decision trees demonstrated moderate accuracy but were prone to overfitting. However, the findings of Alie et al. [53] show that decision trees outperformed several ML models, including random forest, SVM, and logistic regression. While logistic regression outperformed SVM and decision tree, it performed slightly worse than random forest. In contrast to the findings of Chingombe et al. [42], logistic regression outperformed random forest and SVM in predicting HIV risk among the Zimbabwean population in terms of accuracy, recall, and F_1_-score. These discrepancies highlight the ongoing development of ML algorithms. In addition, the nature of data structures and preprocessing can determine the performance of ML algorithms. Despite the disagreements, empirical evidence suggests that random forest is a good model to consider in HIV testing prediction, especially when compared to logistic regression [24,30,45,46].

For many years, public health experts relied on traditional logistic regression in predicting health outcomes. This method is still utilized due to its simplicity, clarity, regulatory friendliness, and measurable feature selection [61]. Despite its effectiveness, traditional logistic regression often misses complex interactions, limiting its accuracy. The emergence of ML tools has gained traction in recent years for showing advantages over traditional statistics [61]. ML exhibits higher predictive power, automatic feature discovery, and flexibility with different data types [61]. A comparison between traditional logistic regression and various ML algorithms shows more advantageous results with random forest for predicting adult sepsis prognosis [62]. Even with modern logistic regression, which possesses better accuracy due to its ML features, it mostly performs worse compared to random forest. Random forest combines several decision trees while minimizing variations in each tree to achieve high predictive accuracy [63]. It is considered a powerful ML tool that is resilient to noise when making predictions [63]. In a benchmark trial with 243 real datasets, random forest yielded higher accuracy in more than 69% of the datasets [64]. These findings, together with empirical evidence [18], are consistent with our results.

Therefore, considering the numerous advantages of random forest, it holds significant potential for designing and implementing HIV testing policies and programs in South Africa. Accurate predictions are essential in optimizing resource allocation toward HIV testing programs, as funding challenges for HIV responses persist. Random forest serves as a vital tool for improving diagnostic accuracy due to its flexibility to analyze various data types, including hospital records and survey data. Leveraging it in predicting high-risk HIV individuals gives South Africa a fighting chance, strengthening trust in testing services while addressing fear of stigma and psychological nuisance associated with reporting false results.

The random forest model consistently achieved the highest predictive performance across all metrics, highlighting its effectiveness in predicting HIV testing behavior among adults in South Africa. These insights highlight the value of ML in public health research, enabling data-driven decision-making to improve HIV testing interventions in South Africa and beyond.

### 4.4. Strengths and Limitations

To the best of our knowledge, this is the first study to apply SML algorithms across the five SABSSM surveys to predict HIV testing among South African adults. The findings show strong predictive performance in both the preliminary and final analyses, with a slightly greater margin in the former. Our study validates shreds of evidence suggesting the use of random forest in HIV risk/testing prediction modeling. A notable strength of this study lies in the implementation of four SML models, which facilitated a comprehensive evaluation of their performance. This methodological approach not only allowed for a thorough comparative analysis but also offered valuable insights into the robustness of predictive outcomes across different modeling frameworks. The use of the SMOOTE technique to address classical data imbalance minimized the overfitting/underfitting of ML models. Furthermore, the methodology was guided by a comprehensive study protocol published as a peer-reviewed article. Importantly, the analysis included a large volume of nationwide survey data, cementing the generalizability of the study findings. Even though the study exclusively uses South African data, SSA countries, especially those with high HIV prevalence, could adopt the methodology to inform HIV testing policies and programs. However, it is important to note that this study’s findings are context-specific and derived from adult population-based surveys in South Africa. Caution should be exercised when applying the models to non-survey datasets or populations in different sociocultural or epidemiological settings. Further validation with external datasets is recommended to test generalizability.

Despite the various strengths highlighted by this study, the study had some limitations. Since the study analyzed secondary data, many missing values were observed during preprocessing. We only included variables with <10 missing values, which excluded potential predictors of HIV testing. Nonetheless, missing values were appropriately treated to enhance meaningful results. Moreover, the SABSSM surveys were conducted through cross-sectional designs, which lack temporality. Hence, future studies should use real-time longitudinal or prospective data to predict HIV testing. In general, biases cannot be completely eradicated in the use of secondary data. Before completing the prediction modeling, we employed risk-for-bias assessment tools coupled with various data preprocessing techniques. The focus on the general population may limit its generalizability to KPs. The mode imputation technique employed might lead to potential bias. This method is used due to its simplicity and effectiveness in categorical variables, and we only included variables with a <10% missing rate. Future studies should explore more robust imputation techniques using random forest, k-nearest neighbors, etc. Some of the ML models, especially decision trees, achieved low accuracy. Future studies should replicate a similar methodology using other ML algorithms that were unexplored in this analysis among various population groups.

## 5. Conclusions

This study shows that ML methods provide effective and reliable strategies for predicting HIV testing behaviors by utilizing sociodemographic and behavioral data collected from repeated adult population-based surveys. The random forest model achieved the highest accuracy (81%), precision (81.6%), F_1_-score (80.3%), and AUC (88.3%), making it the most effective model for predicting HIV testing behavior. These metrics highlight the effectiveness of random forests for spotting individuals who have never tested for HIV across diverse populations. This is crucial for designing targeted and efficient HIV testing interventions in South Africa. The study reveals that knowing a partner’s HIV status, having knowledge of HIV testing sites, being a female, being a younger adult, having high socioeconomic status, and being well-informed about HIV through digital platforms increase the likelihood of being tested for HIV. In contrast, males, older adults, those with a disadvantaged socioeconomic status, and perceiving a low HIV risk negatively influenced HIV testing. The study suggests that leveraging the random forest model enhances data-driven decisions in addressing testing gaps. Hence, policymakers should harness the power of digital platforms to improve awareness of HIV testing and locations, especially among older adults, males, and those living in rural areas. Data privacy and the risk of being stigmatized associated with the use of digital platforms are increasingly concerning. Exploring the feasibility of integrating powerful ML tools, such as random forest, with data encryption and privacy enhancement systems would be valuable.

The findings of this study will serve as baseline evidence for developing HIV testing predictive models using repeated nationwide cross-sectional surveys. Our predictive model should be adapted in designing HIV testing policies and programs to improve precision and resource optimization towards HIV testing programs in South Africa. This forms an integral part of a doctoral study aiming to inform an evidence-based framework for integrating ML in HIV testing in South Africa. The study supports using ML and technology initiatives to address testing policy initiatives focused on awareness, training, media engagement, skill enhancement, employment, accessibility, infrastructure upgrades, shareholder engagement, and targeting high-risk individuals for HIV testing. By implementing these recommendations, South Africa can improve HIV testing uptake and reach the UNAIDS 2030 goal of ending AIDS.

## Figures and Tables

**Table 1:** Temporal changes in features’ predictive power.

SABSSM 2002	Import. Score	SABSSM 2005	Import. Score	SABSSM 2008	Import. Score	SABSSM 2012	Import. Score	SABSSM 2017	Import. Score
Partner’s HIV status	0.201754	Know a place for HIV test	0.077605	Marital status	0.096796	Listen to the radio	0.087542	Listen to the radio	0.112291
Education	0.081740	Drink alcohol	0.076342	Employment	0.087478	Age at first sex	0.079105	Watch TV	0.088266
Age at first sex	0.070158	Contraceptive method	0.073367	Education	0.084785	Circumcised	0.072534	Know a place for HIV test	0.073747
Race	0.065496	Age at first sex	0.065641	Marry a person with HIV	0.084004	Watch TV	0.063809	Circumcised	0.072203
Listen to the radio	0.062167	Race	0.057460	Know a place for HIV test	0.068554	Know a place for HIV test	0.063278	HIV/AIDS can be cured	0.060573
HIV/AIDS can be cured	0.060044	Watch TV	0.056781	Race	0.065472	Reduce HIV risk (few partners)	0.055459	Employment	0.060473
Watch TV	0.049006	Education	0.055873	Circumcised	0.061372	HIV/AIDS can be cured	0.055211	Internet use	0.059864
Ever used a condom	0.042891	Number of children	0.054949	Have a male sex partner	0.058284	Internet use	0.053298	Marital status	0.054407
HIV causes AIDS	0.042692	Marital status	0.051283	Can AIDS be cured?	0.057135	Race	0.053141	Race	0.049284
Ever being married	0.041670	Age in years	0.045837	Where healthcare is obtained	0.052770	Had sex (past 12 months)	0.051005	Ever had sex	0.047184

Note: SABSSM, South African National HIV Prevalence, Incidence, Behavior and Communication Survey; HIV, Human Immunodeficiency Virus; AIDS, Acquired Immunodeficiency Syndrome; TV, television.

**Table 2:** Prevalence of HIV testing from the five SABSSM surveys.

Dataset	*n*	Ever Tested for HIV n (%)
SABSSM 2002	6228	1627 (26.12)
SABSSM 2005	14,285	4816 (33.71)
SABSSM 2008	11,833	6222 (52.58)
SABSSM 2012	24,263	16,133 (66.49)
SABSSM 2017	35,071	26,674 (76.05)

*n*, frequency; %, percentage.

**Table 3:** Distribution of HIV Testing by participants’ sociodemographic information.

Sociodemographic Data	Prevalence of HIV Testing				
	SABSSM 2002 *n* (%)	SABSSM 2005 *n* (%)	SABSSM 2008 *n* (%)	SABSSM 2012 *n* (%)	SABSSM 2017 *n* (%)
Age Category (Years)					
25–34	480 (29.5)	-	1570 (25.3)	4161 (25.8)	7165 (26.9)
35–44	-	1320 (27.4)	-	-	-
Sex					
Female	936 (57.5)	3073 (63.8)	4039 (64.9)	9797 (60.7)	16,704 (62.6)
Race					
African	705 (43.3)	2398 (49.8)	3618 (58.1)	9380 (58.1)	19,754 (74.1)
Province					
Kwazulu-Natal	396 (24.3)	-	1185 (19.0)	1948 (12.1)	8660 (32.5)
Gauteng	-	1044 (21.7)	-	-	-
Geographical location					
Urban	1040 (63.9)	3888 (80.7)	4731 (76.0)	11,416 (70.8)	15,269 (57.2)
Education					
Attended school	-	-		15,383 (95.4)	24,441 (91.6)
Secondary education	1050 (64.5)	1602 (33.3)	4131 (66.4)	-	
Marital status					
Married	1112 (68.3)	2595 (53.9)	2617 (42.1)	-	-
Not married	-	-		7991 (49.5)	15,904 (59.6)
Employment					
Employed	938 (57.7)	2403 (49.9)	3066 (49.3)	8430 (52.3)	-
Unemployed	-	-	-	-	14,762 (55.3)

Note: Only variables with the highest frequency were included in the table, and the denominator was the prevalence of HIV testing in each SABSSM survey; *n*, frequency; %, percentage; SABSSM, South African National HIV Prevalence, Incidence, Behavior and Communication Survey.

**Table 4:** Models’ performance comparisons.

Dataset	Machine Learning Algorithms	Accuracy	Precision	Recall	F_1_-Score	AUC	Cross-Validation Averages
SABSSM 2002	DT	0.737977	0.730942	0.735892	0.733408	0.805684	0.737941
	SVM	0.734107	0.767503	0.655756	0.707243	0.813651	0.720663
	RF	0.809840	0.815116	0.791196	0.802978	0.883056	0.791016
	LR	0.727474	0.736462	0.690745	0.712871	0.814104	0.721355
SABSSM 2005	DT	0.676963	0.667910	0.695596	0.681472	0.736676	0.674389
	SVM	0.686615	0.630614	0.891192	0.738594	0.755959	0.678896
	RF	0.718147	0.696009	0.768135	0.730296	0.798992	0.720564
	LR	0.687902	0.657174	0.777202	0.712166	0.767158	0.697719
SABSSM 2008	DT	0.625103	0.622720	0.620661	0.621689	0.683055	0.647926
	SVM	0.625923	0.583240	0.862810	0.696000	0.713818	0.646284
	RF	0.690730	0.671429	0.738017	0.703150	0.759391	0.697988
	LR	0.668581	0.638239	0.766942	0.696697	0.724994	0.670702
SABSSM 2012	DT	0.692164	0.707021	0.666357	0.686087	0.749359	0.684061
	SVM	0.674992	0.656182	0.748299	0.699220	0.736251	0.678753
	RF	0.741024	0.735004	0.761596	0.748064	0.818769	0.736086
	LR	0.683110	0.667595	0.741497	0.702608	0.745709	0.687456
SABSSM 2017	DT	0.720082	0.735167	0.675219	0.703919	0.790495	0.717503
	SVM	0.645398	0.591038	0.910302	0.716724	0.728749	0.646345
	RF	0.767911	0.751703	0.789976	0.770365	0.853460	0.765308
	LR	0.673527	0.640410	0.769690	0.699124	0.738322	0.669697

SABSSM, South African National HIV Prevalence, Incidence, Behavior and Communication Survey; DT, decision trees; SVM, support vector machines; RF, random forest; LR, logistic regression; AUC, area under the curve.

**Table 5:** Confusion matrices of the machine learning models.

Dataset	ML Models	True Positive	False Positive	True Negative	False Negative
SABSSM 2002	DT	652	240	683	234
	SVM	581	176	747	305
	RF	701	159	764	185
	LR	612	219	704	274
SABSSM 2005	DT	537	267	515	235
	SVM	688	403	379	84
	RF	593	259	523	179
	LR	600	313	469	172
SABSSM 2008	DT	751	455	773	459
	SVM	1044	746	482	166
	RF	893	437	791	317
	LR	928	526	702	282
SABSSM 2012	DT	2155	893	2279	1079
	SVM	2420	1268	1904	814
	RF	2463	888	2284	771
	LR	2398	1194	1978	836
SABSSM 2017	DT	3395	1223	3952	1633
	SVM	4577	3167	2008	451
	RF	3972	1312	3863	1056
	LR	3870	2173	3002	1158

SABSSM, South African National HIV Prevalence, Incidence, Behavior and Communication Survey; ML, machine learning; DT, decision trees; SVM, support vector machines; RF, random forest; LR, logistic regression.

## Data Availability

The datasets analyzed in this study are not publicly available due to the data sharing conditions of the HSRC that prohibit duplication, resharing, or selling the data without prior approval from the rights holder. The datasets can be made available by request from the HSRC via their website.

## Supplementary Materials

The following information can be downloaded at https://www.mdpi.com/article/10.3390/tropicalmed10060167/s1: Table S1: List of selected variables for the HIV testing predictive model; Table S2: Performance metrics of preliminary analysis; Table S3: The most consistent predictors of HIV testing across the five SABSSM surveys; Figure S1: Correlation analysis; Figure S2: Performance metrics of preliminary analysis; Figure S3: Class distribution of HIV testing before and after SMOTE of the five SABSSM datasets.

## Abbreviations

The following abbreviations are used in this study:

ADASYN	Adaptive Synthetic Sampling
AI	Artificial Intelligence
AIDS	Acquired Immunodeficiency Syndrome
ART	Antiretroviral Therapy
AUC-ROC	Area Under Curve–Receiver Operating Characteristics
ENN	Edited Nearest Neighbors
HIV	Human Immunodeficiency Virus
HSCR	Human Science Research Council
KPs	Key Populations
ML	Machine learning
MSM	Men who have Sex with Men
NSP	National Strategic Plan
PII	Personally Identifiable Information
PLWHA	People Living with HIV/AIDS
POPIA	Protection of Personal Information Act
SABSSM	The South African National HIV Prevalence, Incidence, Behavior, and Communication Survey
SML	Supervised Machine Learning
SMOTE	Synthetic Minority Over-sampling Technique
SMOTEENN	Synthetic Minority Over-sampling Technique and Edited Nearest Neighbors
SSA	Sub-Saharan Africa
SVM	Support Vector Machine
UML	Unsupervised Machine Learning
UNAIDS	The Joint United Nations Program on HIV/AIDS
WHO	World Health Organization
XGBoost	Extreme Gradient Boosting
