# Supplementary material for: The Application of Machine Learning Algorithms to Predict HIV Testing Using Evidence from the 2002–2017 South African Adult Population-Based Surveys: An HIV Testing Predictive Model
Source: Trop Med Infect Dis. 2025 Jun 14;10(6):167. doi: 10.3390/tropicalmed10060167 (PMC12197452; doi:10.3390/tropicalmed10060167)
Supplement: Supplementary file 1 [file tropicalmed-10-00167-s001.zip › Table_S1_List_of _Selected_Variables.pdf]

Table S1. List of selected variables for the HIV testing predictive model.

This table consists of the selected variables from the five SABSSM datasets after the data was cleaned, missing variables were treated, and significant predictors were selected in the preprocessing stage (Table S1).

**Variable**	**Variable Definition**	**SABSSM 2002**	**SABSSM 2005**	**SABSSM 2008**	**SABSSM 2012**	**SABSSM 2017**
ever_hivtest	Have you ever had an HIV test?	The outcome variable for all five datasets				
age_cat	Age groups of the respondents	✓	✓	✓	✓	✓
sex	Sex of the respondents	X	X	✓	✓	✓
race	Race	✓	✓	✓	✓	✓
province	Province	✓	✓	✓	✓	✓
geotypye	Geographical location	✓	✓	✓	✓	✓
ever_school	Have you ever attended school?	X	X	X	✓	✓
highest_education	What is the highest level of education you obtained?	✓	✓	✓	X	X
currently_working	Do you currently work?	✓	X	X	X	X
employment	How would you describe your present employment situation?	✓	✓	✓	✓	✓
ever_married	Have you ever been married?	✓	X	X	X	X
marital_status	What is your current marital status?	X	✓	✓	✓	✓
income_last_month	Did you receive any income from any source last month?	X	X	X	✓	✓
ever_sex	Have you ever had sexual intercourse?	✓	✓	✓	✓	✓
age_first_sex	How old were you when you had sex for the first time?	✓	✓	X	X	X
number_children	Number of children	X	✓	X	X	X
sexually_active	Sexually active in the last 12 months	X	✓	X	X	X
current_sex_partners	How many sexual partners do you currently have?	X	✓	X	X	X
num_sexual_partners	Number of sexual partners	X	X	✓	X	X
sex_partner_last12m	Number of sexual partners in the last 12 months.	✓	X	X	X	X
female_sex_partners	Number of female sexual partners	X	X	✓	X	X
male_sex_partners	Number of male sexual partners	X	X	✓	X	X
ever_condom_use	Have you ever used a condom?	✓	X	✓	X	X
condom_first_sex	Did you use a condom the first time you had sex?	X	✓	✓	✓	✓
get_condom	Should you need a condom, is it possible to get one?	✓	X	X	X	X
prev_hiv_last_sex	Have you ever used a condom?	X	X	✓	X	X
ever_drink_alcohol	Have you ever had a drink containing alcohol?	✓	✓	✓	✓	✓
alcohol_freq	How often did you have a drink containing alcohol in the last 12 months	X	✓	X	X	X
disability	Do you have a disability?	X	X	✓	✓	✓
hypertension	Diagnosed with hypertension/ high blood pressure?	X	✓	X	✓	✓
diabetes	Diagnosed with diabetes?	X	✓	X	✓	✓

hosp_past_12months	In the past 12 months, have you been hospitalized for any illness?	X	X	X	✓	✓
place_obtain_healthcare	Where do you usually obtain health care?	X	X	✓	✓	✓
physically_forced_sex	Have you been physically forced to have sex against your will in the past year?	X	✓	✓	X	X
listen_radio	How often do you listen to the radio?	✓	X	X	✓	✓
watch_tv	How often do you watch TV?	✓	✓		✓	✓
use_internet	How often do you use the internet?	✓	✓	X	✓	✓
attendhiv_edu	In the past 12 months, did you attend a play, dialogue, or education on HIV/AIDS?	X	X	X	✓	✓
hivaids_info_tv	HIV causes AIDS	✓	X	X	X	X
condom_everytime_prevhiv	Can a person reduce the risk of getting HIV by using a condom every time he/she has sex?	X	X	X	✓	✓
can_aids_cured	Can AIDS be cured?	✓	✓	✓	✓	✓
hiv_cause_aids	HIV causes AIDS	✓	X	X	X	X
partner_hiv_status	Do you know your partner's HIV status?	✓	X	X	X	X
marry_person_hivaids	HIV/AIDS information – TV programs	X	X	✓	X	X
resp_hiv_prev	Who should be more responsible for preventing new HIV infections?	X	✓	X	X	X
transmit_hiv_inject	Is it possible to transmit HIV by sharing needle injections?	X	✓	X	X	X
transmit_hiv_unprt_anal_sex	Is it possible to transmit HIV by having an unprotected anal sex?	X	✓	X	X	X
transmit_hiv_unprt_vagnl_sex	Is it possible to transmit HIV by having an unprotected vaginal sex?	X	✓	X	X	X
male_circumcision_prevhiv	Can medical male circumcision reduce the risk of HIV infection in males?	X	X	X	✓	✓
circumcised	Have you been circumcised?	X	X	✓	X	X
household_member_hiv	Has anyone in your household ever been diagnosed with HIV/AIDS?	X	✓	X	X	X
perceived_hivrisk	On a scale of 1 to 4, how would you rate yourself in terms of risk of becoming infected with HIV?	X	X	✓	✓	✓
know_place_hivtest	Do you know of a place nearby where you can get an HIV test?	X	✓	✓	✓	✓

✓significant ($p<0.05$) variables; X variable was not present in the data set or variable excluded due to missing >10% or variable excluded due to $p>0.05$ from the Chi-square test; HIV/AIDS, human immunodeficiency virus/acquired immunodeficiency syndrome; TV, television.
