# Supplementary material for: The Application of Machine Learning Algorithms to Predict HIV Testing Using Evidence from the 2002–2017 South African Adult Population-Based Surveys: An HIV Testing Predictive Model
Source: Trop Med Infect Dis. 2025 Jun 14;10(6):167. doi: 10.3390/tropicalmed10060167 (PMC12197452; doi:10.3390/tropicalmed10060167)
Supplement: Supplementary file 1 [file tropicalmed-10-00167-s001.zip › Table_S2_Performance Metrics_Preliminary_Analysis.pdf]

Table S2. Performance metrics

Data set	Machine learning algorithms	Accuracy	Precision	Recall	F-1 score	AUC	Cross-validation averages
**SABSSM 2002**	**DT**	0.796573	0.792325	0.792325	0.792325	0.854228	0.780373
	**SVM**	0.829187	0.901252	0.731377	0.807477	0.908519	0.824603
	**RF**	0.838032	0.832772	0.837472	0.835115	0.915500	0.830131
	**LR**	0.831951	0.856618	0.788939	0.821387	0.910334	0.826123
**SABSSM 2005**	**DT**	0.707851	0.708115	0.700777	0.704427	0.760689	0.674878
	**SVM**	0.725869	0.708937	0.760363	0.733750	0.817023	0.731344
	**RF**	0.759331	0.745074	0.783679	0.763889	0.835287	0.752579
	**LR**	0.733591	0.724311	0.748705	0.736306	0.819865	0.732469
**SABSSM 2008**	**DT**	0.667350	0.661017	0.676860	0.668844	0.713722	0.662083
	**SVM**	0.664889	0.623043	0.822314	0.708942	0.732968	0.687627
	**RF**	0.710829	0.688012	0.763636	0.723854	0.778836	0.719633
	**LR**	0.691961	0.662651	0.772727	0.713468	0.754817	0.702605
**SABSSM 2012**	**DT**	0.724477	0.743939	0.692641	0.717374	0.786120	0.720865
	**SVM**	0.760849	0.756171	0.776747	0.766321	0.842570	0.762353
	**RF**	0.777865	0.770057	0.798392	0.783968	0.859182	0.778549
	**LR**	0.777552	0.748694	0.841991	0.792607	0.853372	0.779369
**SABSSM 2017**	**DT**	0.771319	0.815830	0.704892	0.756314	0.828715	0.764334
	**SVM**	0.758342	0.819180	0.667280	0.735469	0.813250	0.760130
	**RF**	0.799656	0.854664	0.725408	0.784749	0.870305	0.801211
	**LR**	0.750132	0.776016	0.708048	0.740476	0.824110	0.751291

SABSSM, South African National HIV Prevalence, Incidence, Behavioral and Communication Survey; DT, Decision Trees; SVM, Support Vector Machines; RF, Random Forest; LR, Logistic Regression; AUC, Area under curve
