# Supplementary material for: The Application of Machine Learning Algorithms to Predict HIV Testing Using Evidence from the 2002–2017 South African Adult Population-Based Surveys: An HIV Testing Predictive Model
Source: Trop Med Infect Dis. 2025 Jun 14;10(6):167. doi: 10.3390/tropicalmed10060167 (PMC12197452; doi:10.3390/tropicalmed10060167)
Supplement: Supplementary file 1 [file tropicalmed-10-00167-s001.zip › Table_S3_Consistent_Predictors_of_HIV_Testing.pdf]

Table S3. Consistent predictors of HIV testing across the five surveys

	SABSSM 2002	SABSSM 2005	SABSSM 2008	SABSSM 2012	SABSSM 2017
Partner's HIV status	✓				
Education	✓	✓	✓		
Race	✓	✓	✓	✓	✓
Age at first sex	✓	✓		✓	
Listen to the radio	✓			✓	✓
Can HIV/AIDS be cured?	✓	✓	✓	✓	✓
Watch TV	✓	✓		✓	✓
Condom use	✓	✓	✓		✓
Male circumcision	✓		✓	✓	✓
Drink alcohol	✓	✓	✓	✓	✓
Marital status	✓	✓	✓	✓	✓
Can HIV cause AIDS?	✓				
Internet use	✓	✓		✓	✓
HIV/AIDS information from TV	✓				
Province	✓		✓		✓
Geographical location	✓	✓	✓	✓	✓
Employment	✓	✓	✓	✓	✓
Age	✓	✓	✓		
Know a place for HIV test		✓	✓	✓	
Contraceptive method		✓			
Number of children		✓			
Pereceived HIV risk		✓	✓		✓
Sex		✓		✓	✓
HIV transmission by unprotected anal sex		✓			
Responsible for HIV prevention		✓			
Marry a person with HIV/AIDS			✓		
Where healthcare is obtained			✓	✓	✓
Male sex artners			✓		
Female sex partners			✓		
HIV preventions at last sex			✓		
Physically forced to have sex			✓		
Had sex in the past 12months				✓	
Reduce HIV risk by having few partners				✓	
Ever had sex				✓	

Note: SBASSM, South African HIV Prevalence, Incidence, Behaviour and Communication Survey; ✓ indicates the presence of a significant predictor.
