# Supplementary figures and images for: The Application of Machine Learning Algorithms to Predict HIV Testing Using Evidence from the 2002–2017 South African Adult Population-Based Surveys: An HIV Testing Predictive Model
Source: Trop Med Infect Dis. 2025 Jun 14;10(6):167. doi: 10.3390/tropicalmed10060167 (PMC12197452; doi:10.3390/tropicalmed10060167)

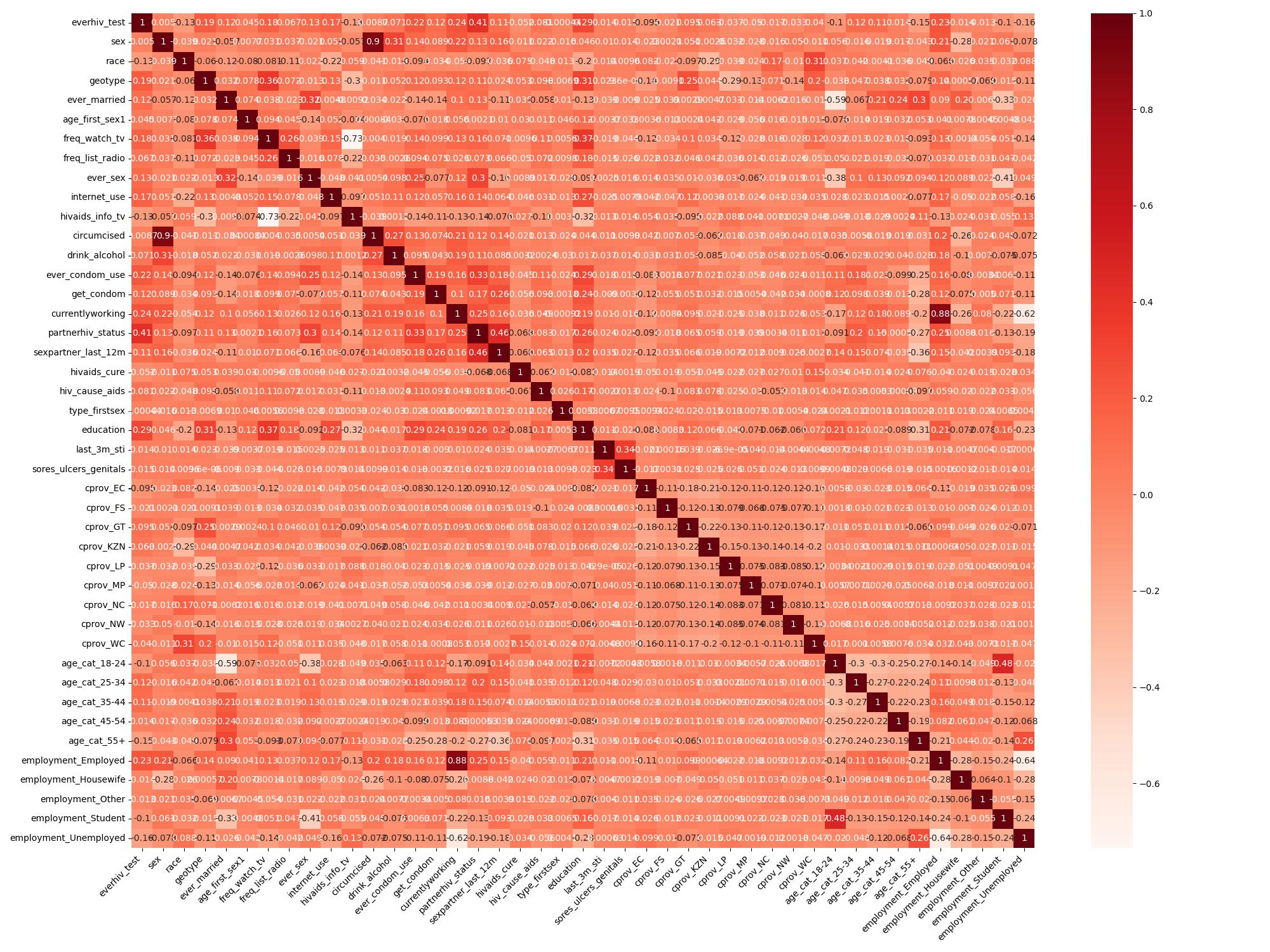

Supplement: Supplementary file 1 [file tropicalmed-10-00167-s001.zip › Figure_S1_Correlation analysis/cor_2002.jpg]

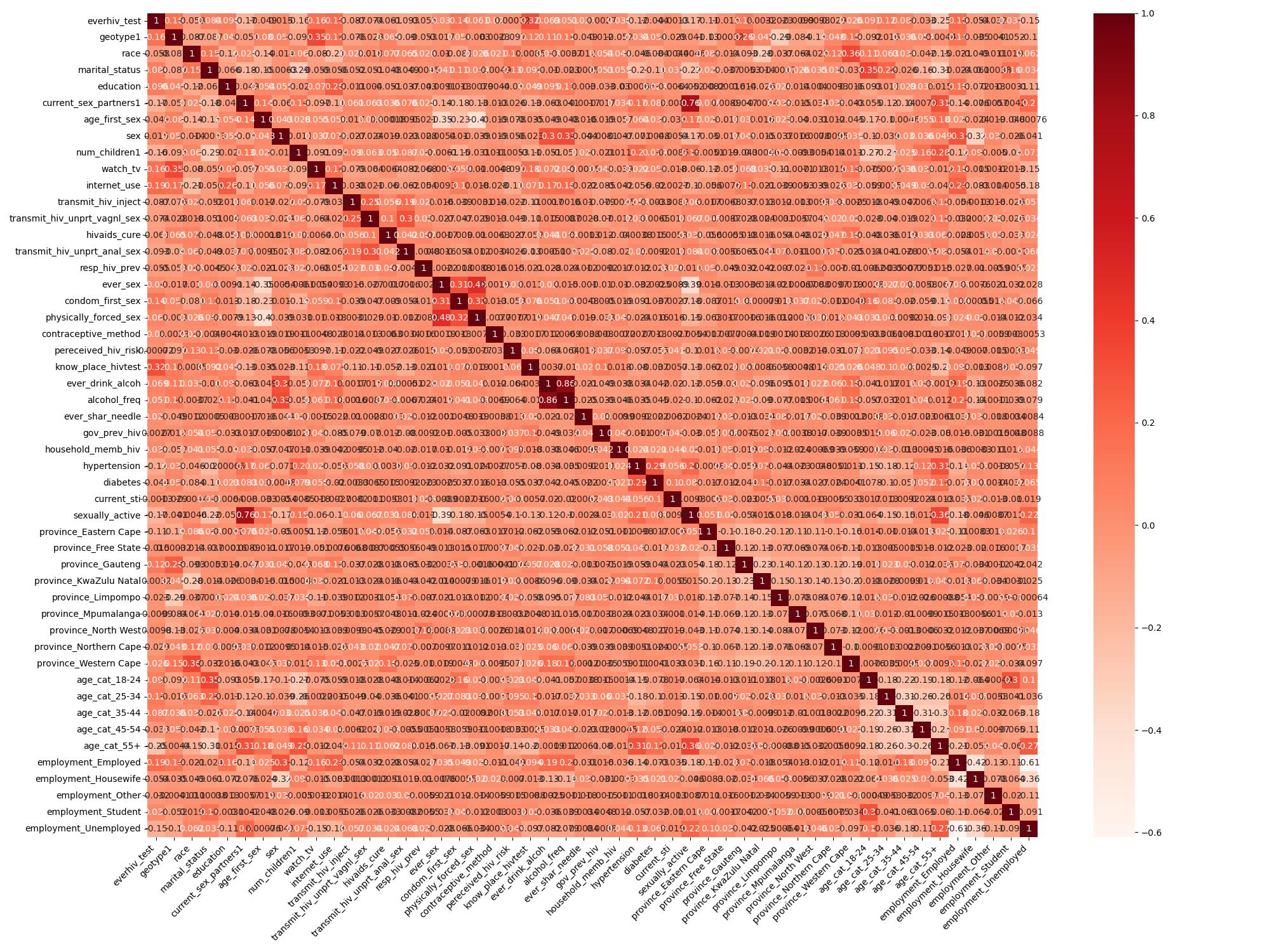

Supplement: Supplementary file 1 [file tropicalmed-10-00167-s001.zip › Figure_S1_Correlation analysis/cor_2005.jpg]

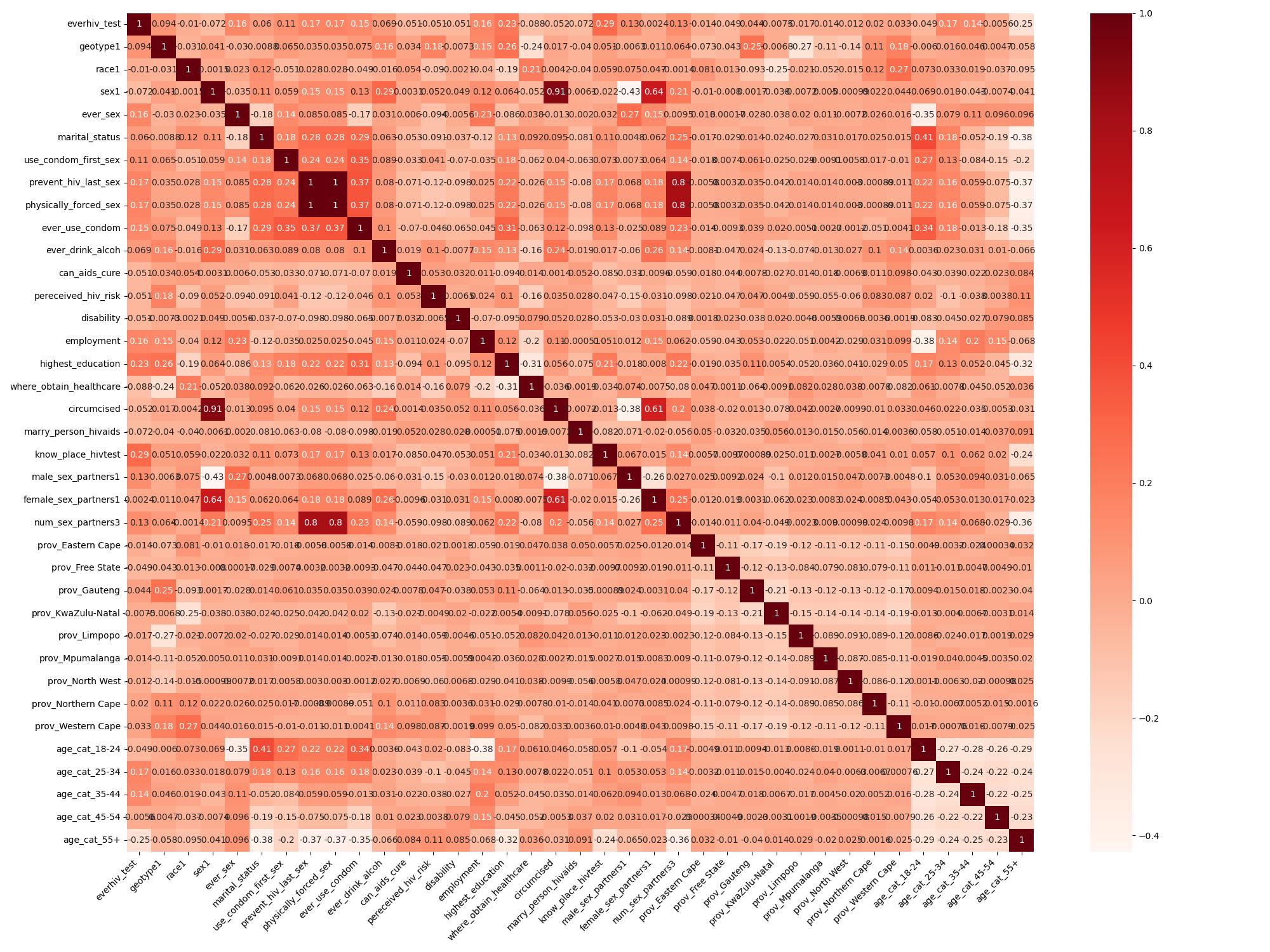

Supplement: Supplementary file 1 [file tropicalmed-10-00167-s001.zip › Figure_S1_Correlation analysis/cor_2008.jpg]

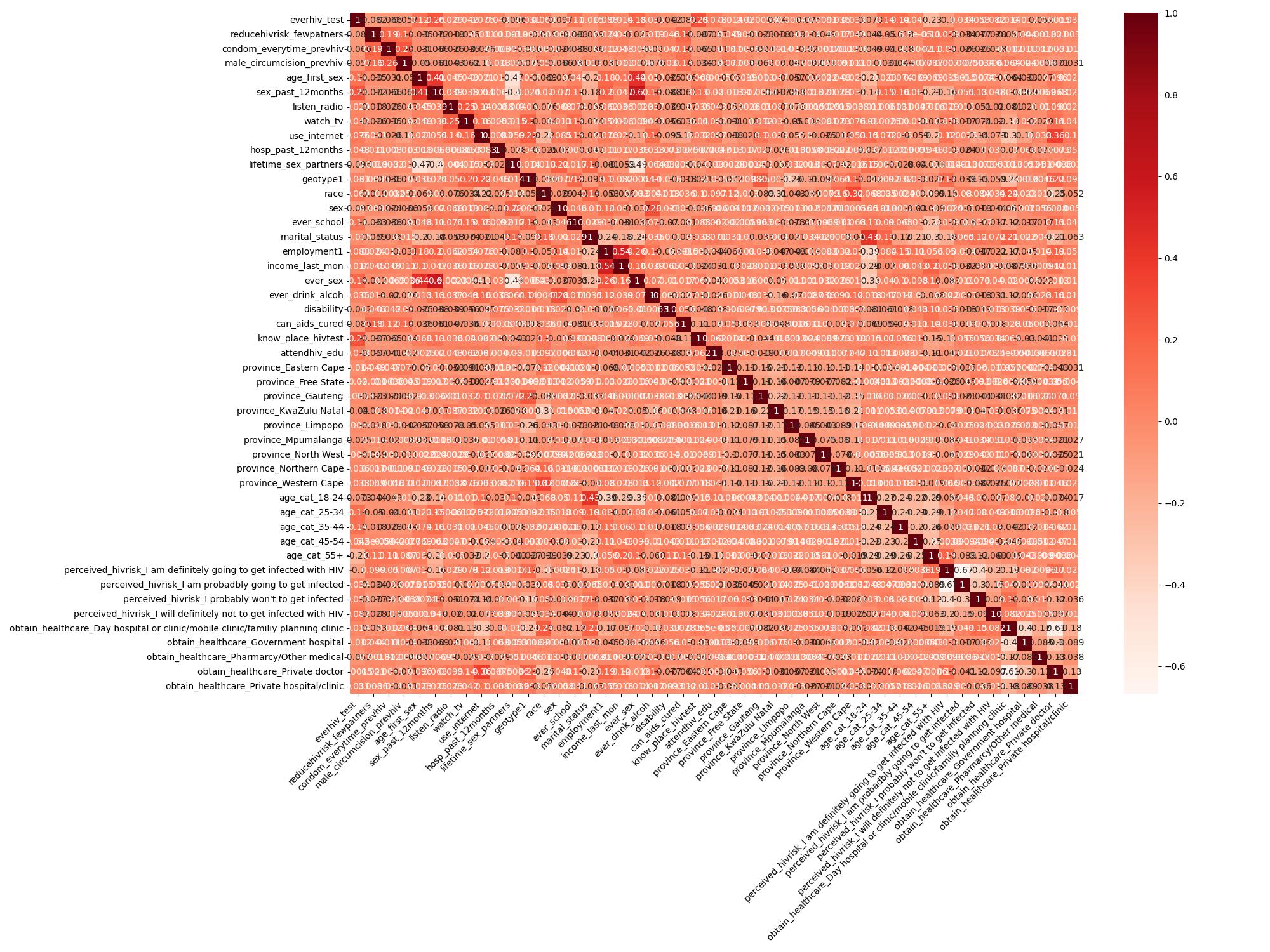

Supplement: Supplementary file 1 [file tropicalmed-10-00167-s001.zip › Figure_S1_Correlation analysis/cor_2012.jpg]

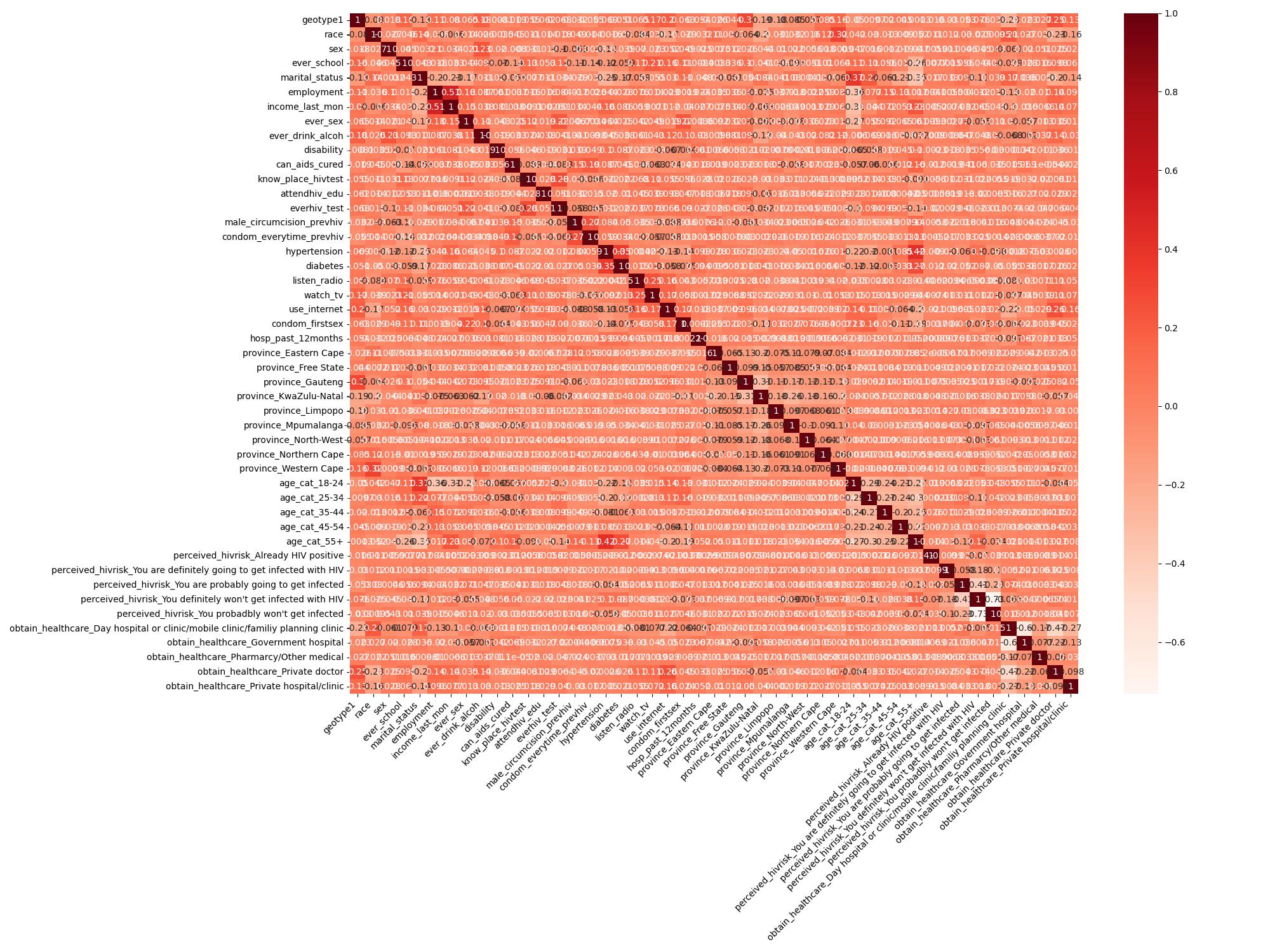

Supplement: Supplementary file 1 [file tropicalmed-10-00167-s001.zip › Figure_S1_Correlation analysis/cor_2017.jpg]

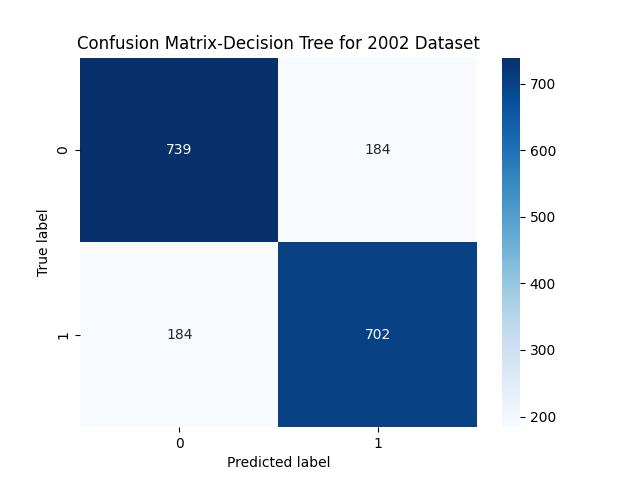

Supplement: Supplementary file 1 [file tropicalmed-10-00167-s001.zip › Figure_S2_Performance_Metrics_Preliminary_Analysis/DT_2002.jpg]

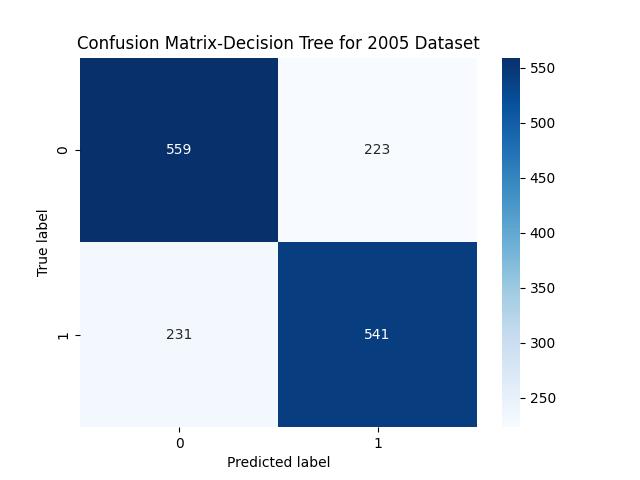

Supplement: Supplementary file 1 [file tropicalmed-10-00167-s001.zip › Figure_S2_Performance_Metrics_Preliminary_Analysis/DT_2005.jpg]

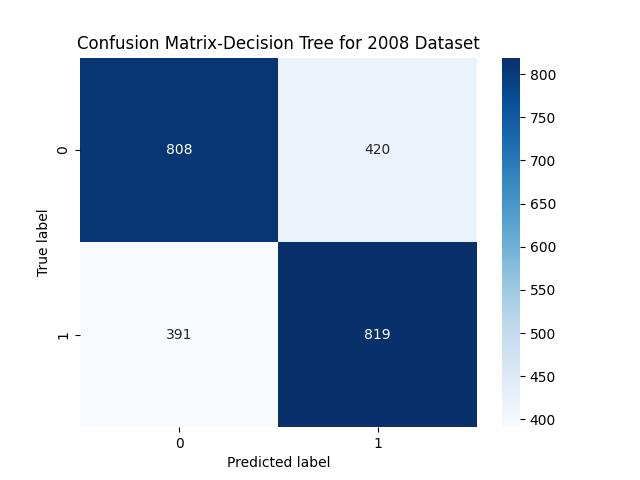

Supplement: Supplementary file 1 [file tropicalmed-10-00167-s001.zip › Figure_S2_Performance_Metrics_Preliminary_Analysis/DT_2008.jpg]

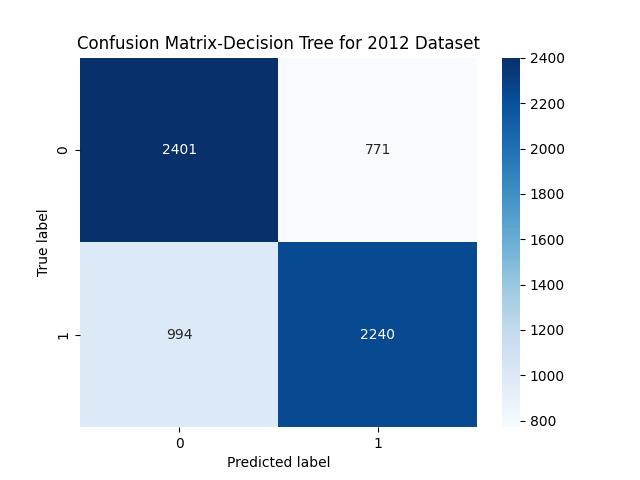

Supplement: Supplementary file 1 [file tropicalmed-10-00167-s001.zip › Figure_S2_Performance_Metrics_Preliminary_Analysis/DT_2012.jpg]

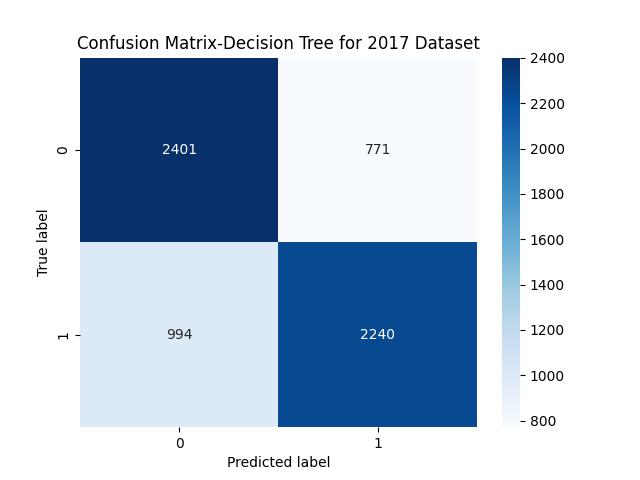

Supplement: Supplementary file 1 [file tropicalmed-10-00167-s001.zip › Figure_S2_Performance_Metrics_Preliminary_Analysis/DT_2017.jpg]

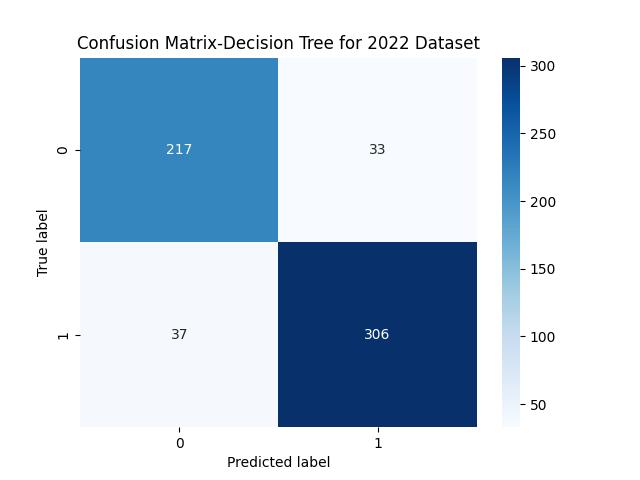

Supplement: Supplementary file 1 [file tropicalmed-10-00167-s001.zip › Figure_S2_Performance_Metrics_Preliminary_Analysis/DT_2022.jpg]

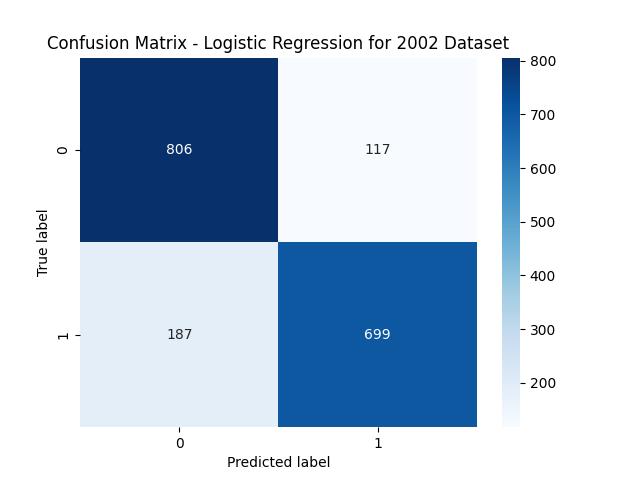

Supplement: Supplementary file 1 [file tropicalmed-10-00167-s001.zip › Figure_S2_Performance_Metrics_Preliminary_Analysis/LR-CM_2002.jpg]

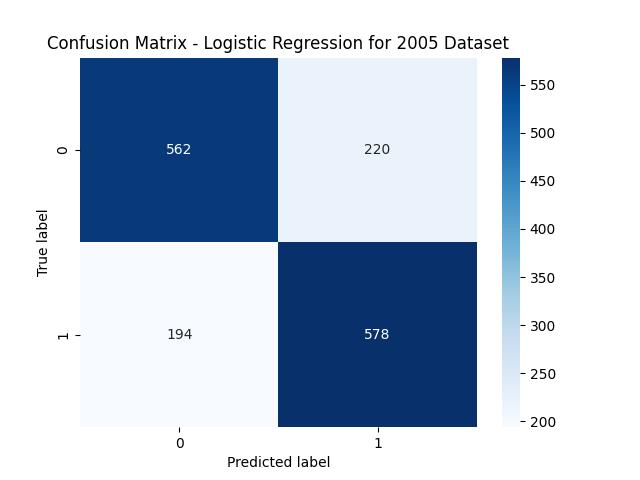

Supplement: Supplementary file 1 [file tropicalmed-10-00167-s001.zip › Figure_S2_Performance_Metrics_Preliminary_Analysis/LR-CM_2005.jpg]

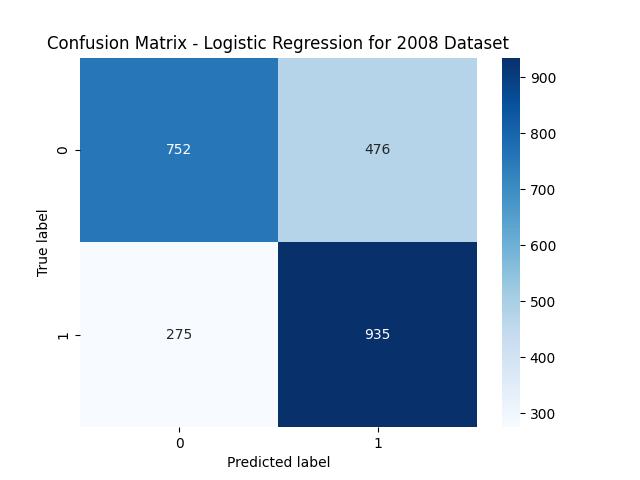

Supplement: Supplementary file 1 [file tropicalmed-10-00167-s001.zip › Figure_S2_Performance_Metrics_Preliminary_Analysis/LR-CM_2008.jpg]

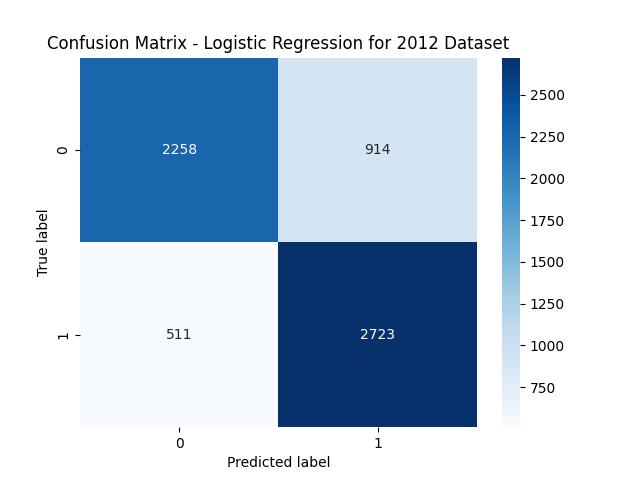

Supplement: Supplementary file 1 [file tropicalmed-10-00167-s001.zip › Figure_S2_Performance_Metrics_Preliminary_Analysis/LR-CM_2012.jpg]

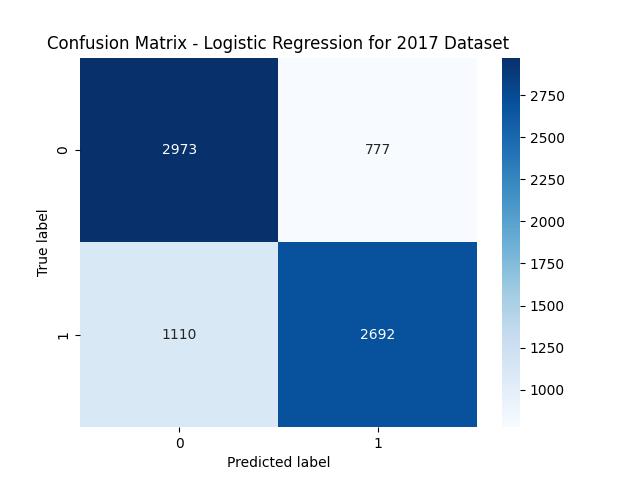

Supplement: Supplementary file 1 [file tropicalmed-10-00167-s001.zip › Figure_S2_Performance_Metrics_Preliminary_Analysis/LR-CM_2017.jpg]

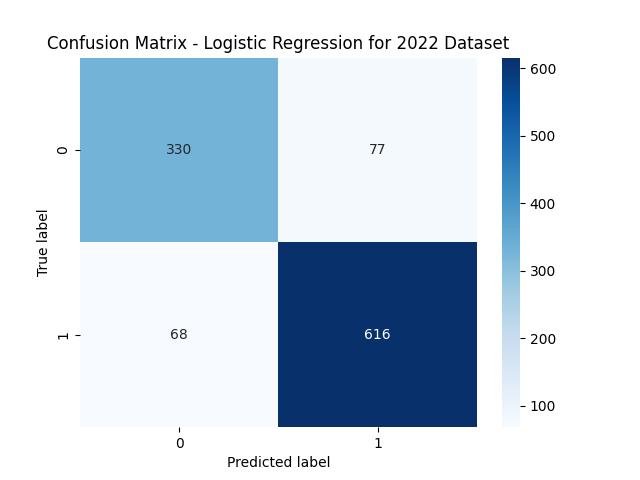

Supplement: Supplementary file 1 [file tropicalmed-10-00167-s001.zip › Figure_S2_Performance_Metrics_Preliminary_Analysis/LR-CM_2022.jpg]

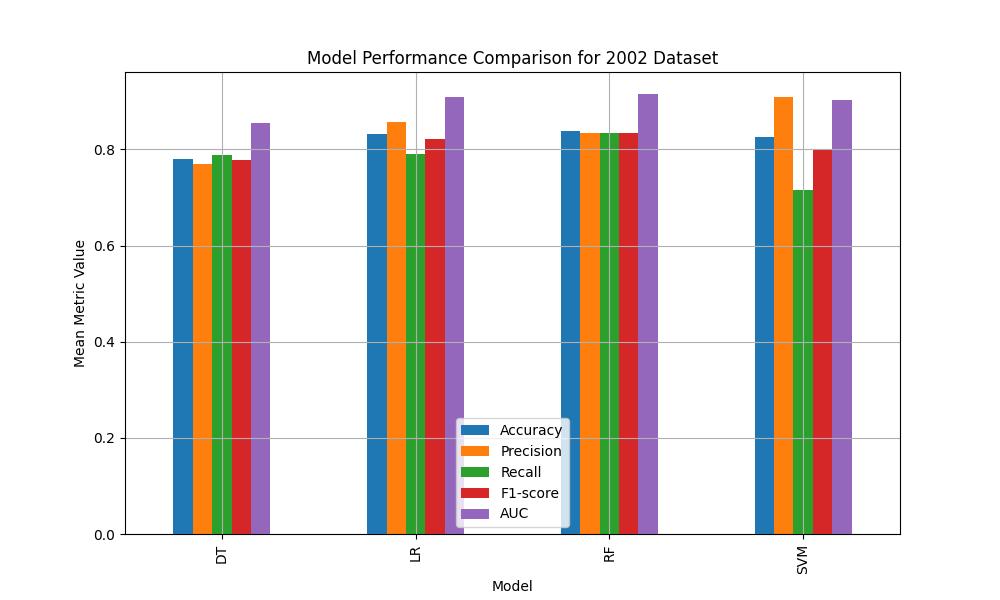

Supplement: Supplementary file 1 [file tropicalmed-10-00167-s001.zip › Figure_S2_Performance_Metrics_Preliminary_Analysis/Model_Performance_Comparison_2002.jpg]

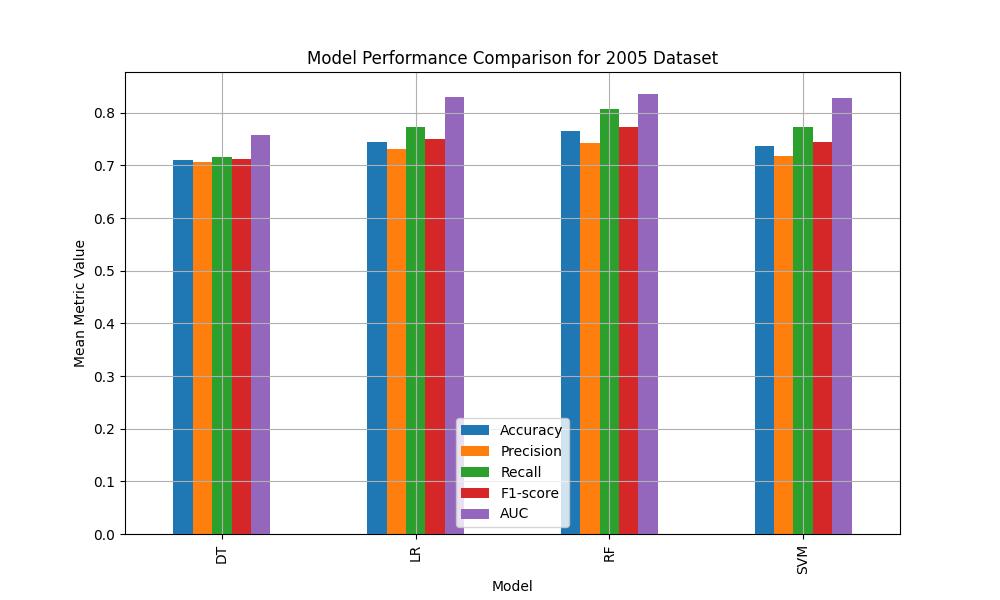

Supplement: Supplementary file 1 [file tropicalmed-10-00167-s001.zip › Figure_S2_Performance_Metrics_Preliminary_Analysis/Model_Performance_Comparison_2005.jpg]

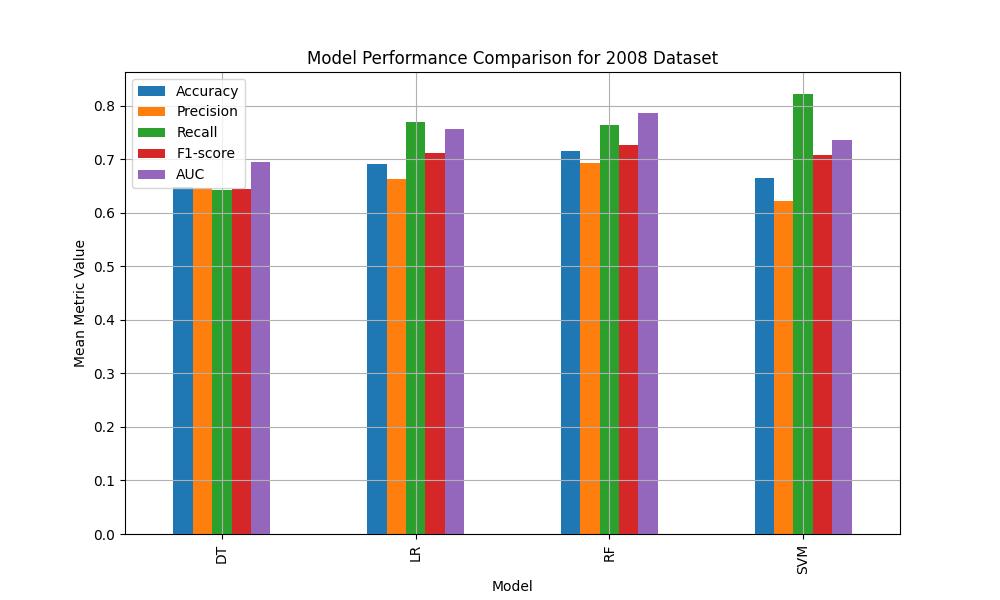

Supplement: Supplementary file 1 [file tropicalmed-10-00167-s001.zip › Figure_S2_Performance_Metrics_Preliminary_Analysis/Model_Performance_Comparison_2008.jpg]

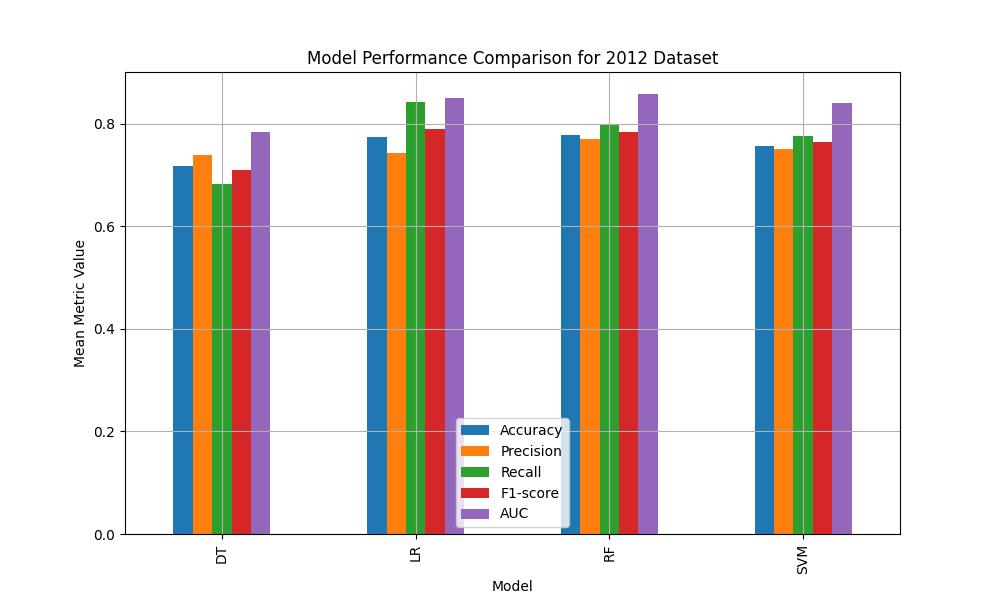

Supplement: Supplementary file 1 [file tropicalmed-10-00167-s001.zip › Figure_S2_Performance_Metrics_Preliminary_Analysis/Model_Performance_Comparison_2012.jpg]

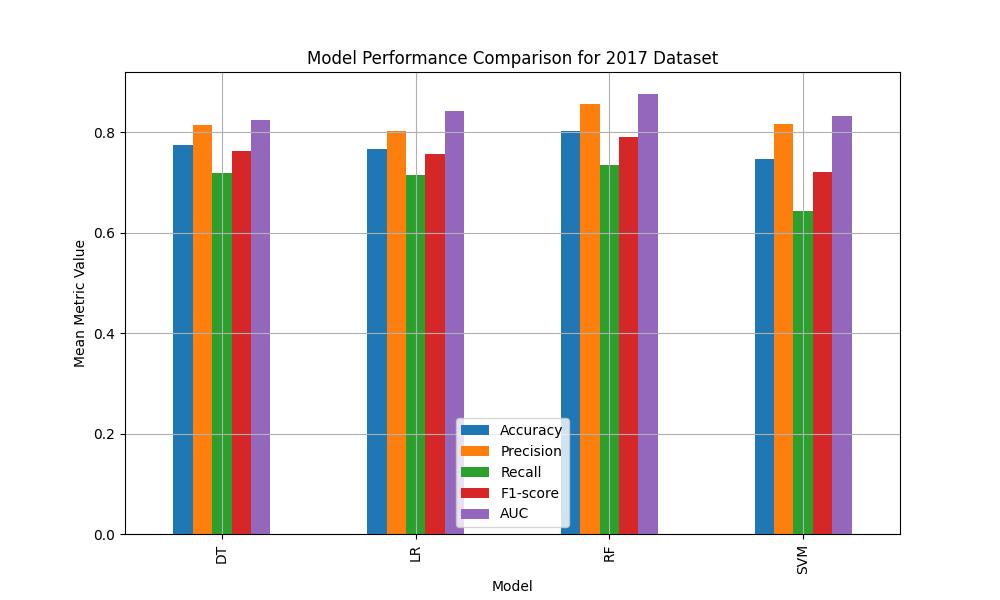

Supplement: Supplementary file 1 [file tropicalmed-10-00167-s001.zip › Figure_S2_Performance_Metrics_Preliminary_Analysis/Model_Performance_Comparison_2017.jpg]

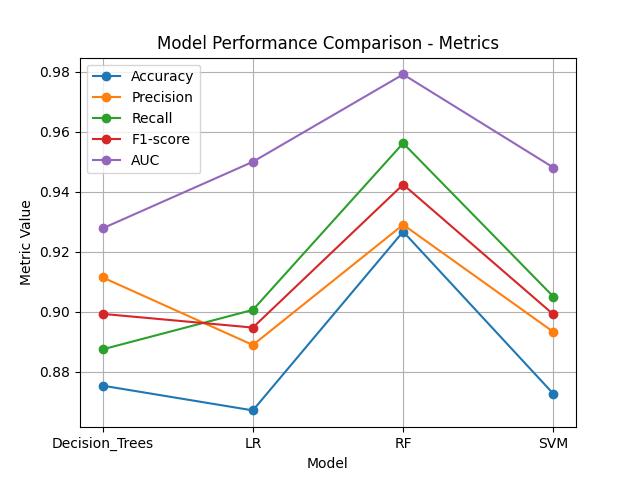

Supplement: Supplementary file 1 [file tropicalmed-10-00167-s001.zip › Figure_S2_Performance_Metrics_Preliminary_Analysis/Model_Performance_Comparison_metric.jpg]

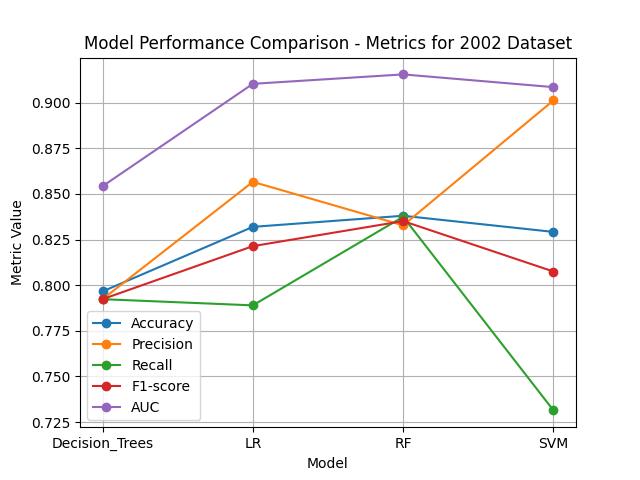

Supplement: Supplementary file 1 [file tropicalmed-10-00167-s001.zip › Figure_S2_Performance_Metrics_Preliminary_Analysis/Model_Performance_Comparison_metric_2002.jpg]

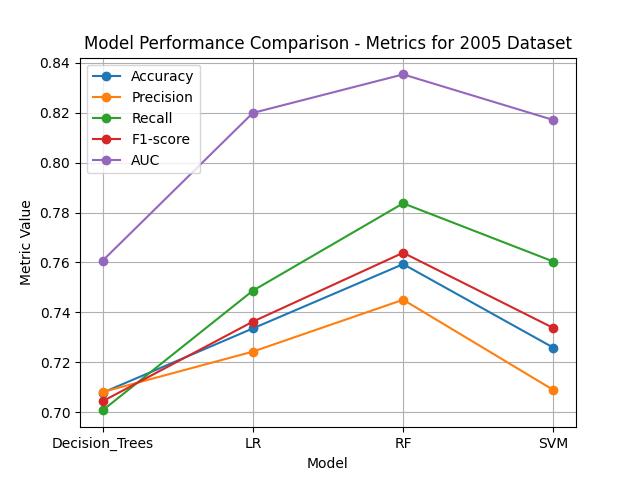

Supplement: Supplementary file 1 [file tropicalmed-10-00167-s001.zip › Figure_S2_Performance_Metrics_Preliminary_Analysis/Model_Performance_Comparison_metric_2005.jpg]

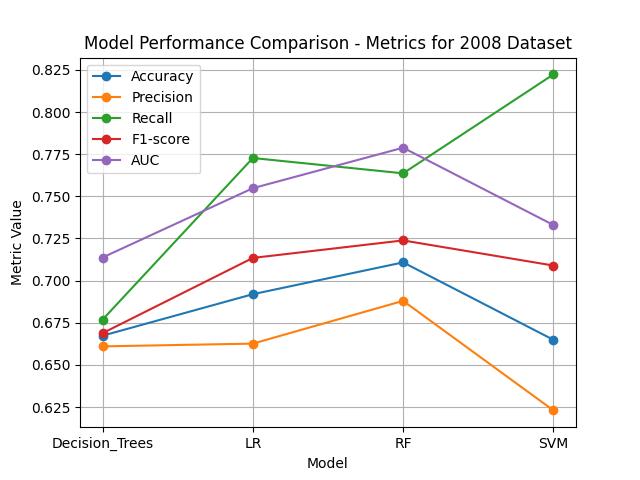

Supplement: Supplementary file 1 [file tropicalmed-10-00167-s001.zip › Figure_S2_Performance_Metrics_Preliminary_Analysis/Model_Performance_Comparison_metric_2008.jpg]

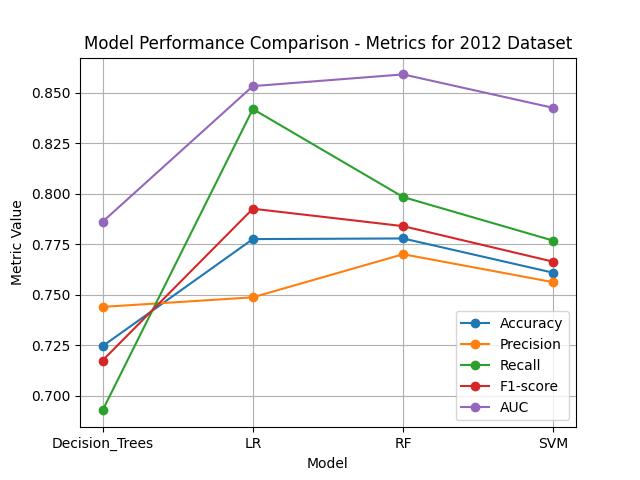

Supplement: Supplementary file 1 [file tropicalmed-10-00167-s001.zip › Figure_S2_Performance_Metrics_Preliminary_Analysis/Model_Performance_Comparison_metric_2012.jpg]

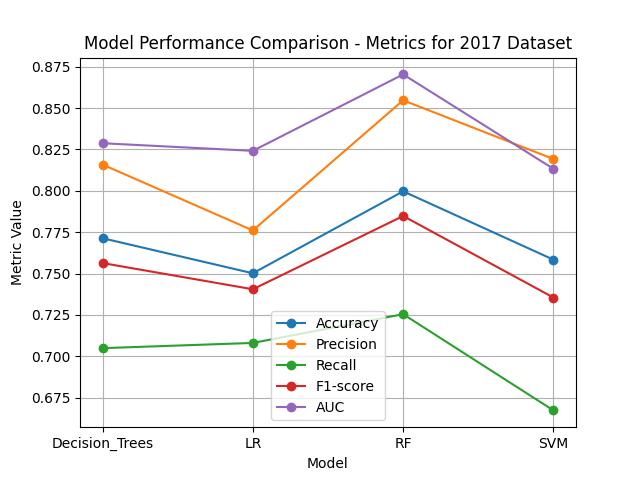

Supplement: Supplementary file 1 [file tropicalmed-10-00167-s001.zip › Figure_S2_Performance_Metrics_Preliminary_Analysis/Model_Performance_Comparison_metric_2017.jpg]

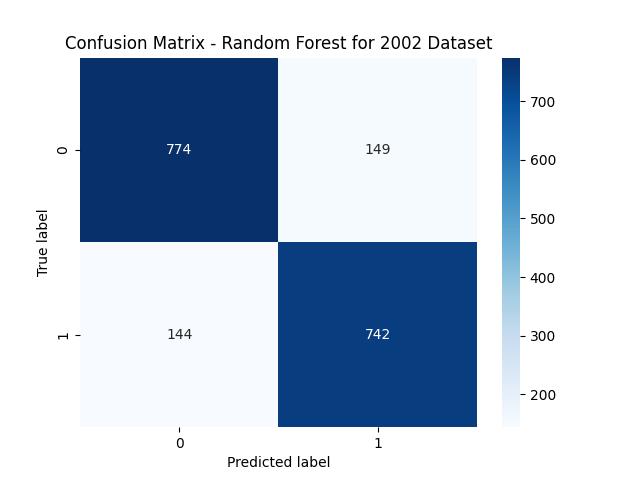

Supplement: Supplementary file 1 [file tropicalmed-10-00167-s001.zip › Figure_S2_Performance_Metrics_Preliminary_Analysis/RF-CM_2002.jpg]

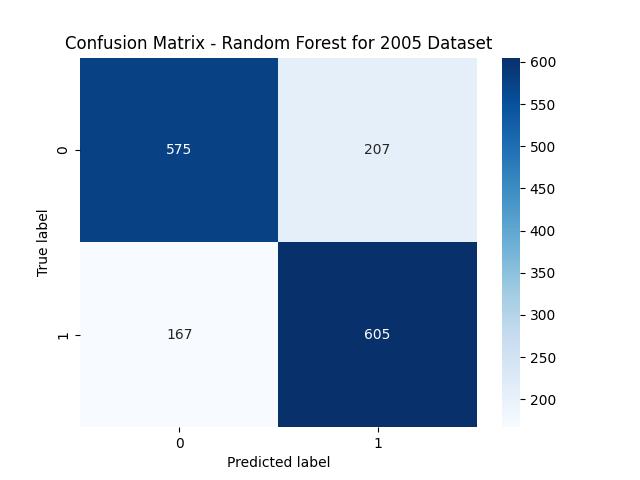

Supplement: Supplementary file 1 [file tropicalmed-10-00167-s001.zip › Figure_S2_Performance_Metrics_Preliminary_Analysis/RF-CM_2005.jpg]

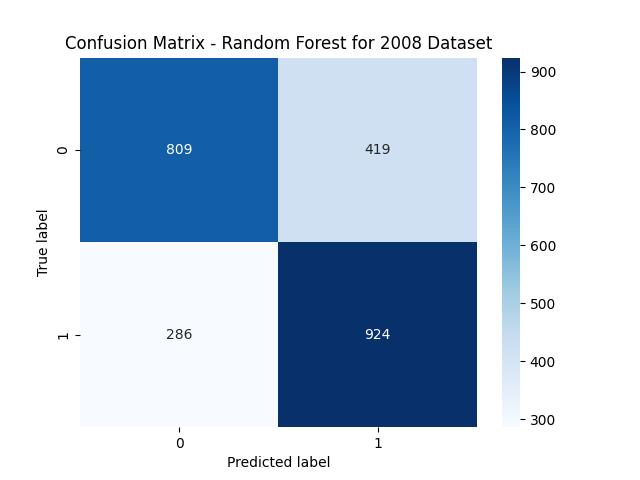

Supplement: Supplementary file 1 [file tropicalmed-10-00167-s001.zip › Figure_S2_Performance_Metrics_Preliminary_Analysis/RF-CM_2008.jpg]

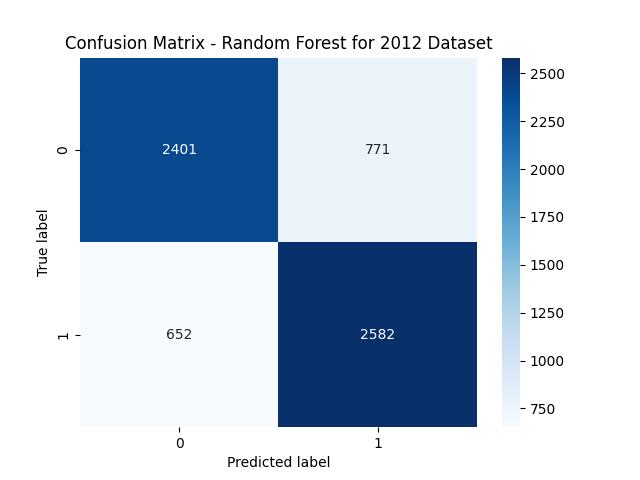

Supplement: Supplementary file 1 [file tropicalmed-10-00167-s001.zip › Figure_S2_Performance_Metrics_Preliminary_Analysis/RF-CM_2012.jpg]

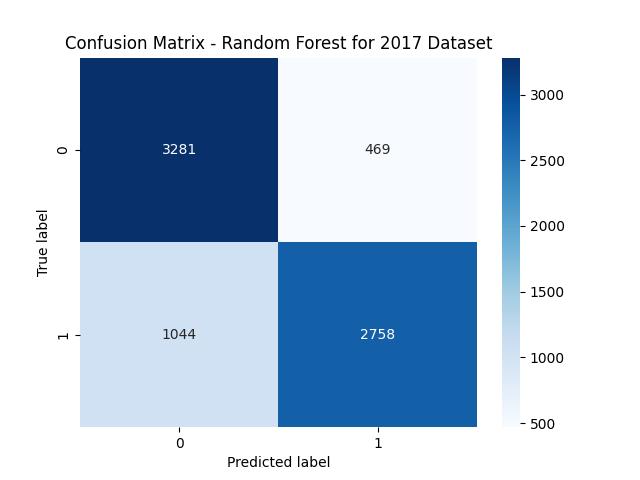

Supplement: Supplementary file 1 [file tropicalmed-10-00167-s001.zip › Figure_S2_Performance_Metrics_Preliminary_Analysis/RF-CM_2017.jpg]

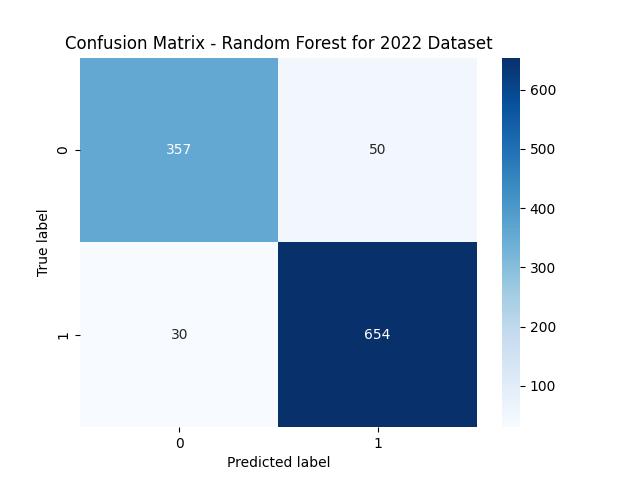

Supplement: Supplementary file 1 [file tropicalmed-10-00167-s001.zip › Figure_S2_Performance_Metrics_Preliminary_Analysis/RF-CM_2022.jpg]

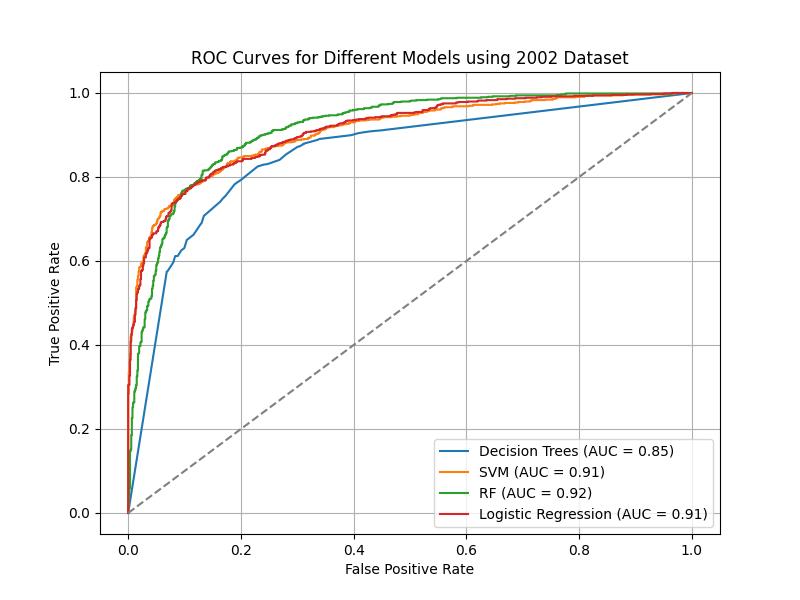

Supplement: Supplementary file 1 [file tropicalmed-10-00167-s001.zip › Figure_S2_Performance_Metrics_Preliminary_Analysis/ROC_2002.jpg]

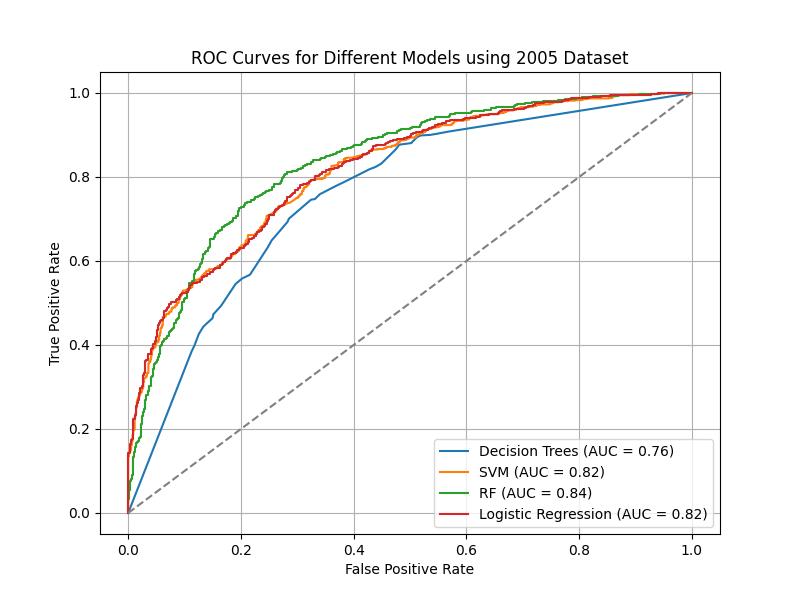

Supplement: Supplementary file 1 [file tropicalmed-10-00167-s001.zip › Figure_S2_Performance_Metrics_Preliminary_Analysis/ROC_2005.jpg]

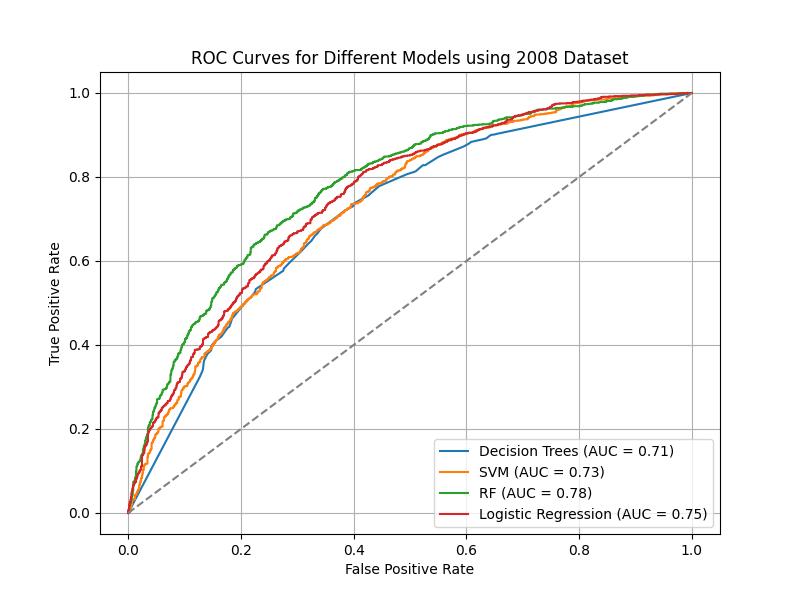

Supplement: Supplementary file 1 [file tropicalmed-10-00167-s001.zip › Figure_S2_Performance_Metrics_Preliminary_Analysis/ROC_2008.jpg]

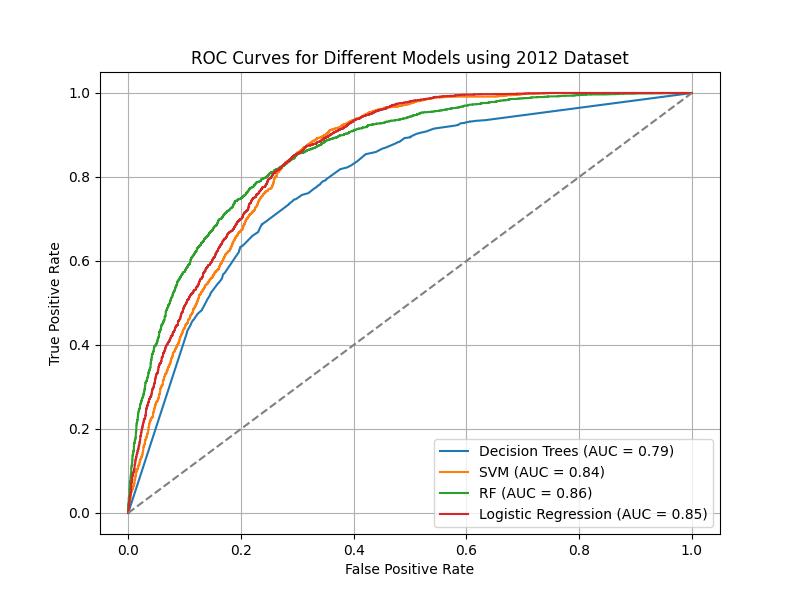

Supplement: Supplementary file 1 [file tropicalmed-10-00167-s001.zip › Figure_S2_Performance_Metrics_Preliminary_Analysis/ROC_2012.jpg]

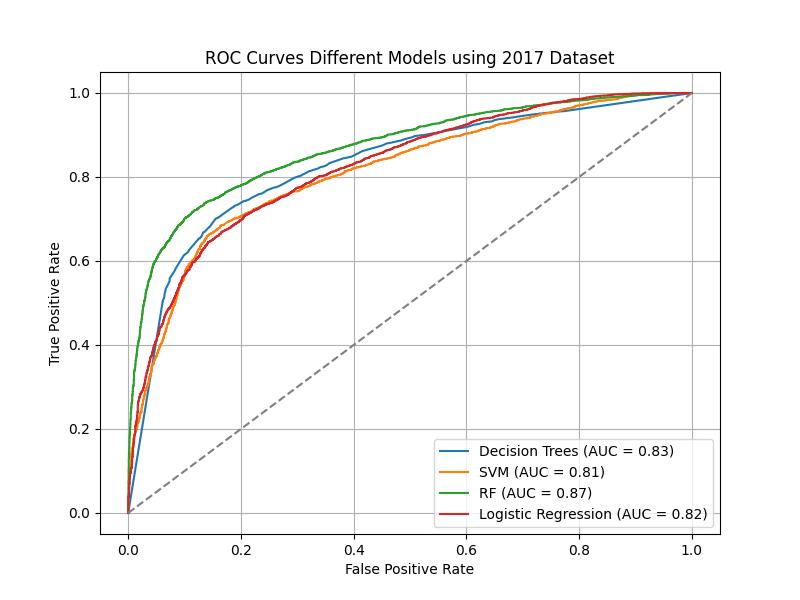

Supplement: Supplementary file 1 [file tropicalmed-10-00167-s001.zip › Figure_S2_Performance_Metrics_Preliminary_Analysis/ROC_2017.jpg]

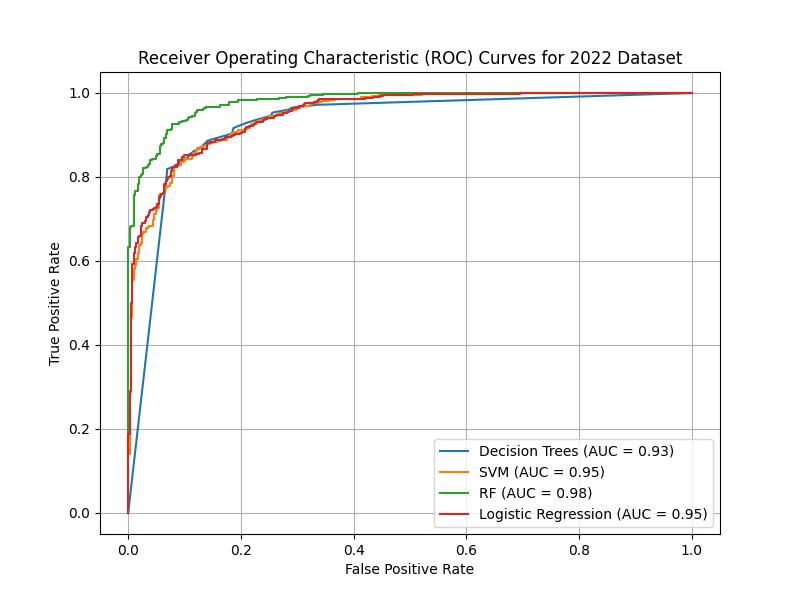

Supplement: Supplementary file 1 [file tropicalmed-10-00167-s001.zip › Figure_S2_Performance_Metrics_Preliminary_Analysis/ROC_2022.jpg]

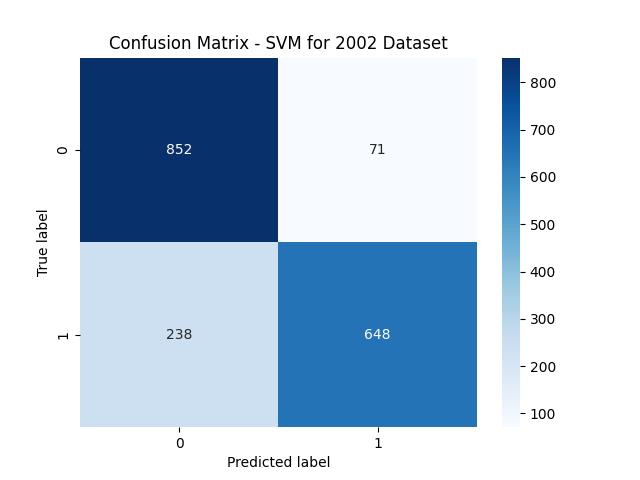

Supplement: Supplementary file 1 [file tropicalmed-10-00167-s001.zip › Figure_S2_Performance_Metrics_Preliminary_Analysis/SVM-CM_2002.jpg]

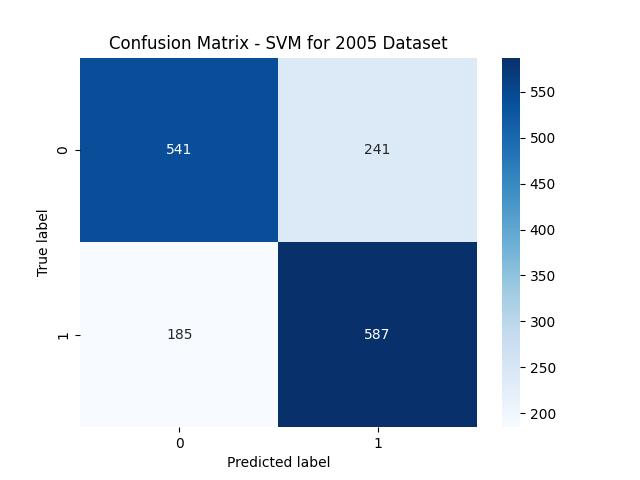

Supplement: Supplementary file 1 [file tropicalmed-10-00167-s001.zip › Figure_S2_Performance_Metrics_Preliminary_Analysis/SVM-CM_2005.jpg]

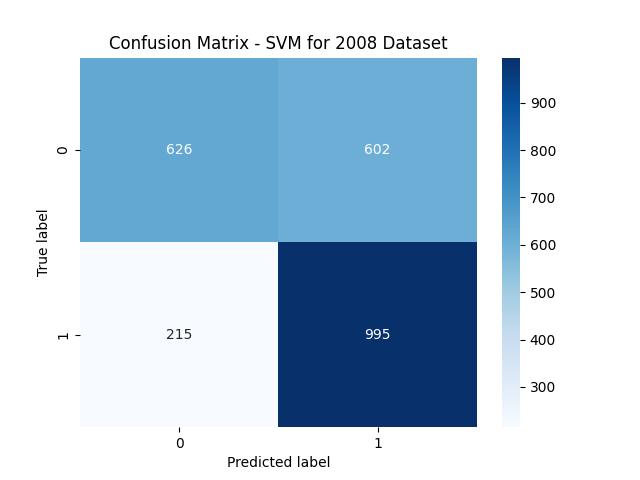

Supplement: Supplementary file 1 [file tropicalmed-10-00167-s001.zip › Figure_S2_Performance_Metrics_Preliminary_Analysis/SVM-CM_2008.jpg]

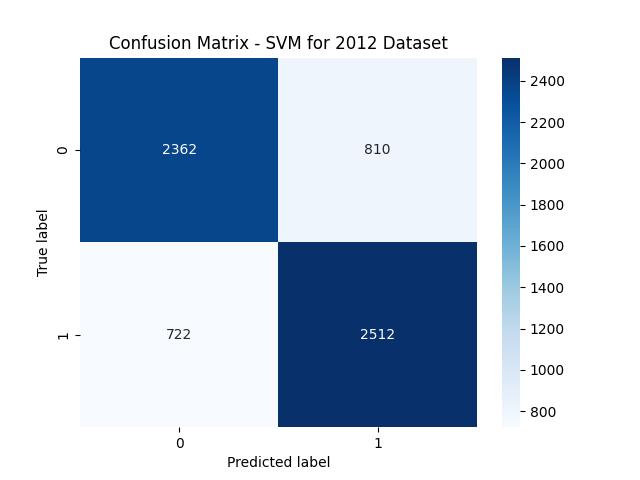

Supplement: Supplementary file 1 [file tropicalmed-10-00167-s001.zip › Figure_S2_Performance_Metrics_Preliminary_Analysis/SVM-CM_2012.jpg]

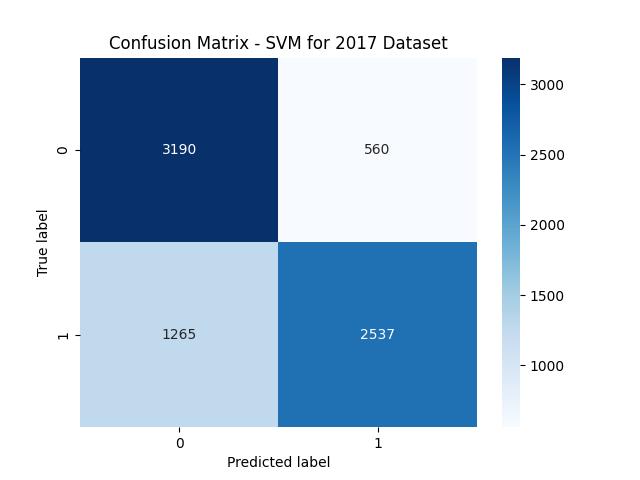

Supplement: Supplementary file 1 [file tropicalmed-10-00167-s001.zip › Figure_S2_Performance_Metrics_Preliminary_Analysis/SVM-CM_2017.jpg]

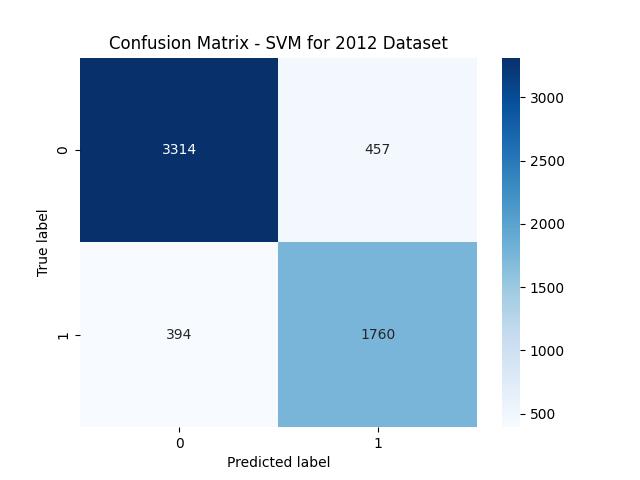

Supplement: Supplementary file 1 [file tropicalmed-10-00167-s001.zip › Figure_S2_Performance_Metrics_Preliminary_Analysis/SVM-CM_2022.jpg]

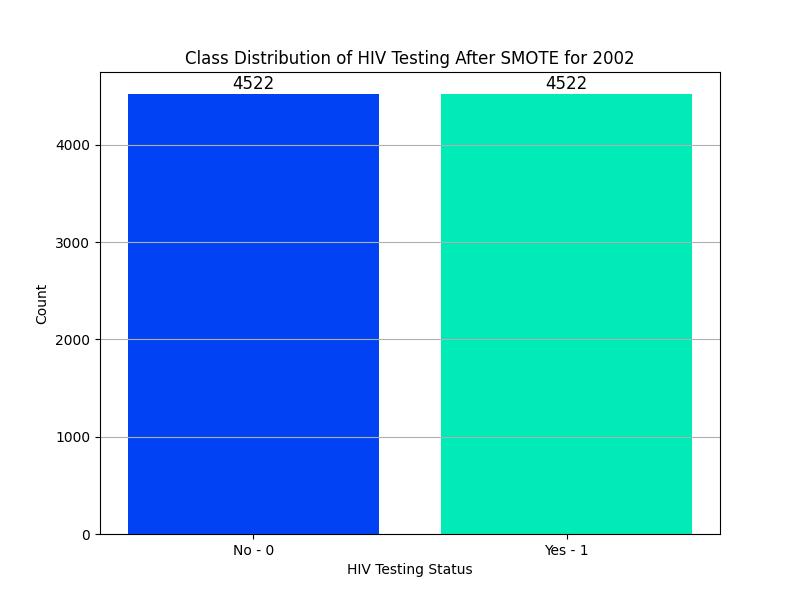

Supplement: Supplementary file 1 [file tropicalmed-10-00167-s001.zip › Figure_S3_Classical_Distribution of HIV Testing_Before_After_SMOTE/balance_after_smoteenn_2002.jpg]

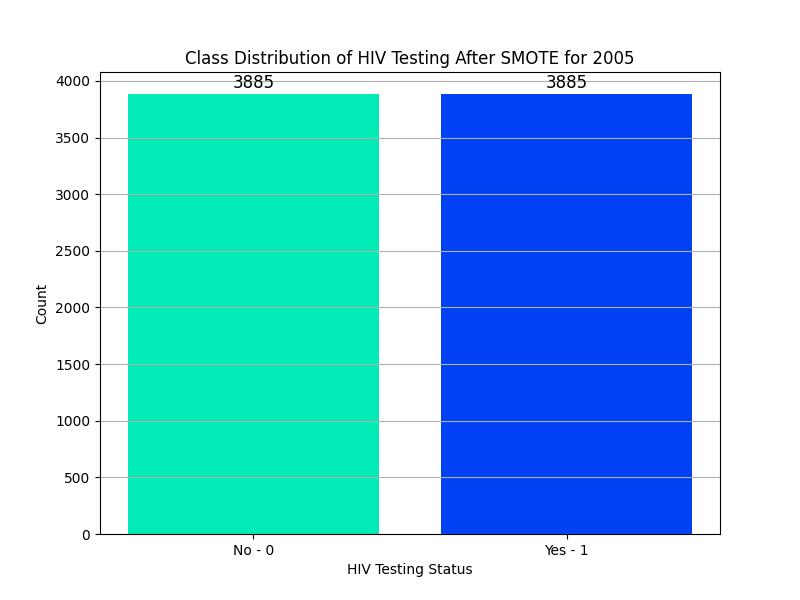

Supplement: Supplementary file 1 [file tropicalmed-10-00167-s001.zip › Figure_S3_Classical_Distribution of HIV Testing_Before_After_SMOTE/balance_after_smoteenn_2005.jpg]

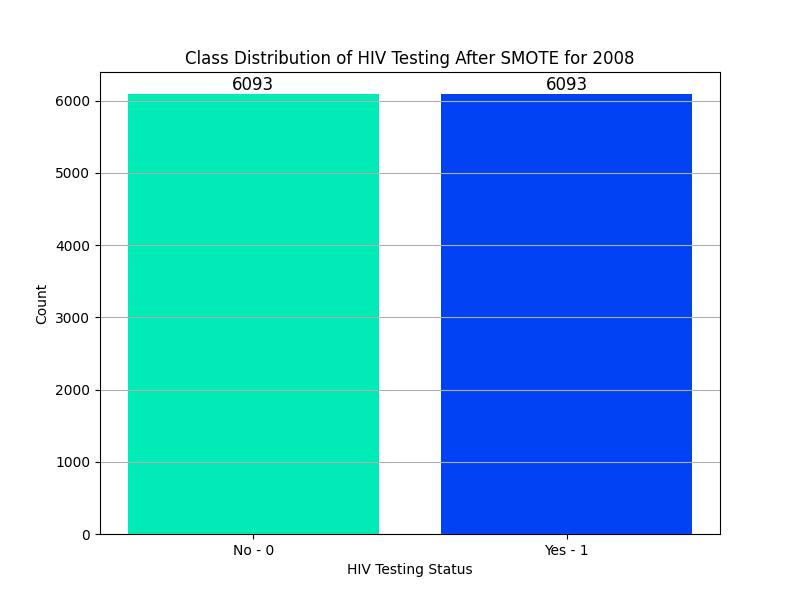

Supplement: Supplementary file 1 [file tropicalmed-10-00167-s001.zip › Figure_S3_Classical_Distribution of HIV Testing_Before_After_SMOTE/balance_after_smoteenn_2008.jpg]

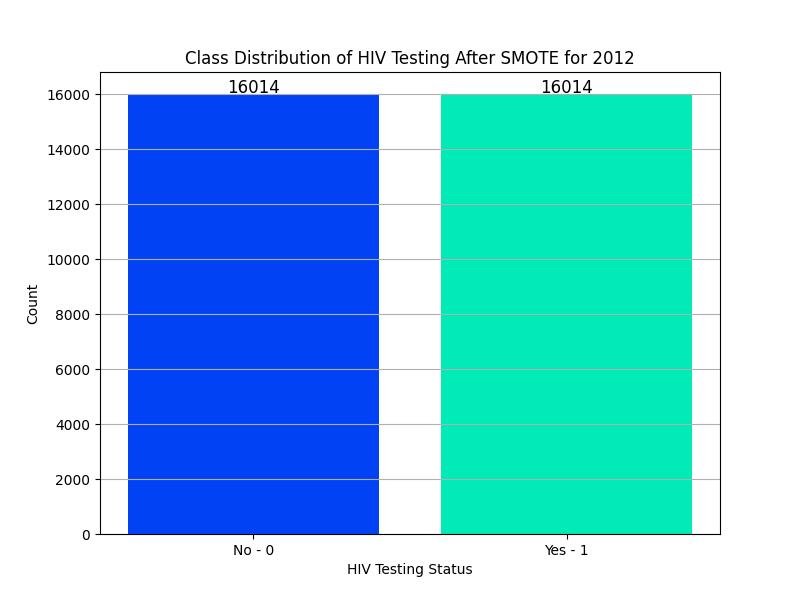

Supplement: Supplementary file 1 [file tropicalmed-10-00167-s001.zip › Figure_S3_Classical_Distribution of HIV Testing_Before_After_SMOTE/balance_after_smoteenn_2012.jpg]

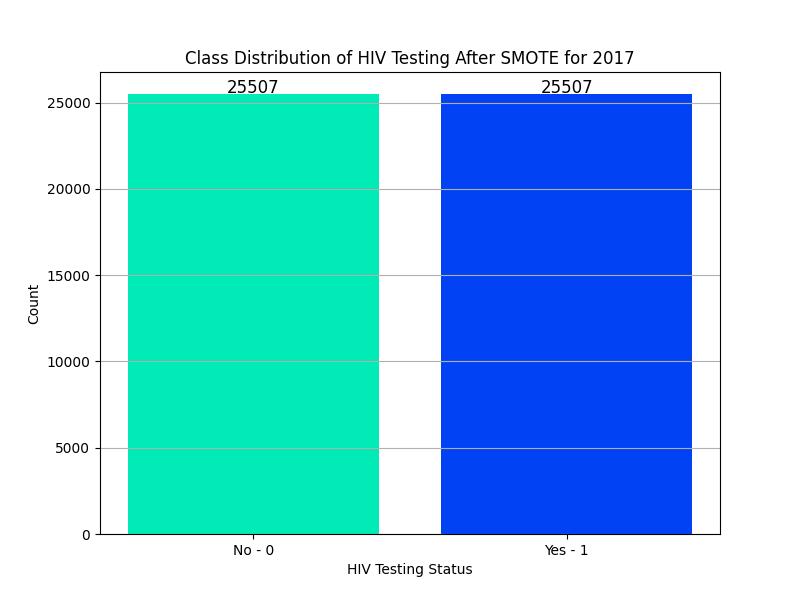

Supplement: Supplementary file 1 [file tropicalmed-10-00167-s001.zip › Figure_S3_Classical_Distribution of HIV Testing_Before_After_SMOTE/balance_after_smoteenn_2017.jpg]

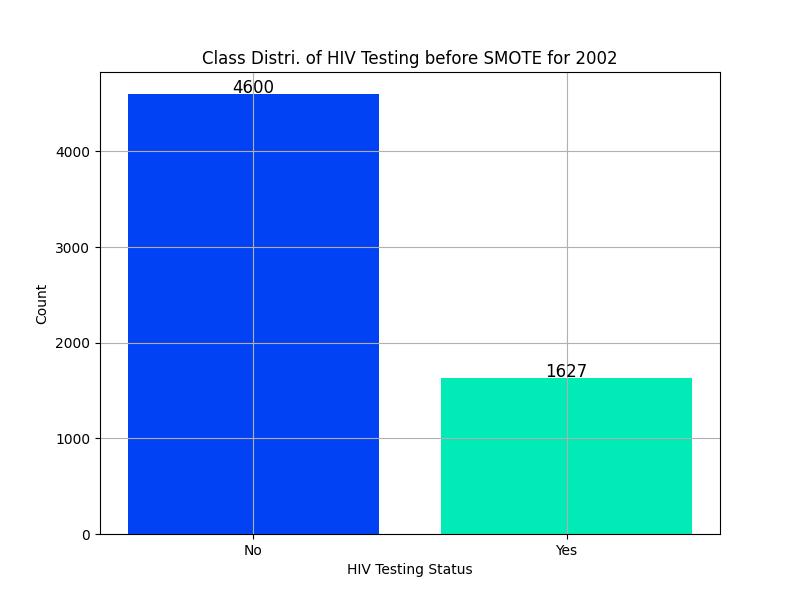

Supplement: Supplementary file 1 [file tropicalmed-10-00167-s001.zip › Figure_S3_Classical_Distribution of HIV Testing_Before_After_SMOTE/balance_before_smoteenn_2002.jpg]

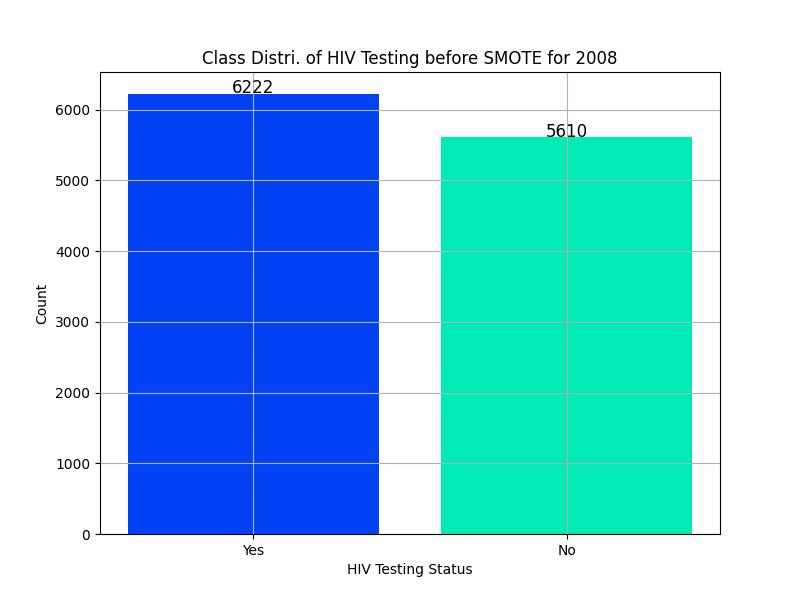

Supplement: Supplementary file 1 [file tropicalmed-10-00167-s001.zip › Figure_S3_Classical_Distribution of HIV Testing_Before_After_SMOTE/balance_before_smoteenn_2008.jpg]

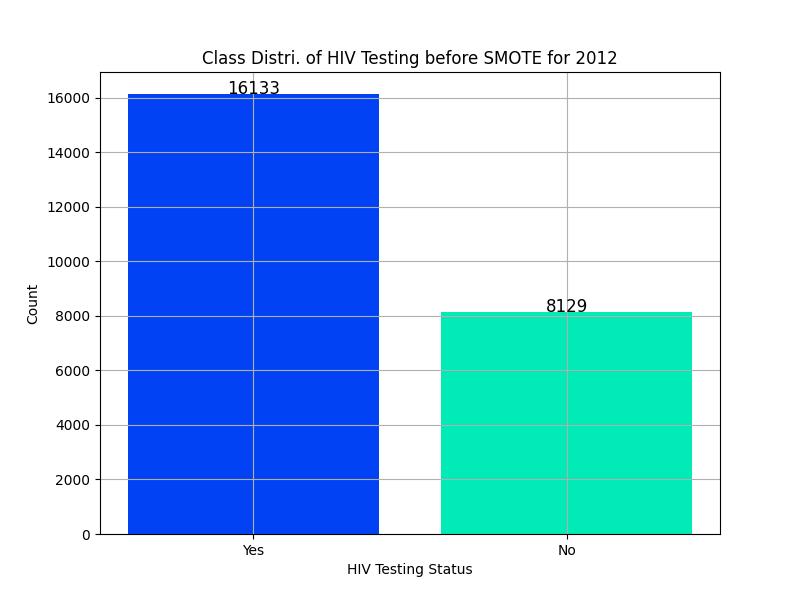

Supplement: Supplementary file 1 [file tropicalmed-10-00167-s001.zip › Figure_S3_Classical_Distribution of HIV Testing_Before_After_SMOTE/balance_before_smoteenn_2012.jpg]

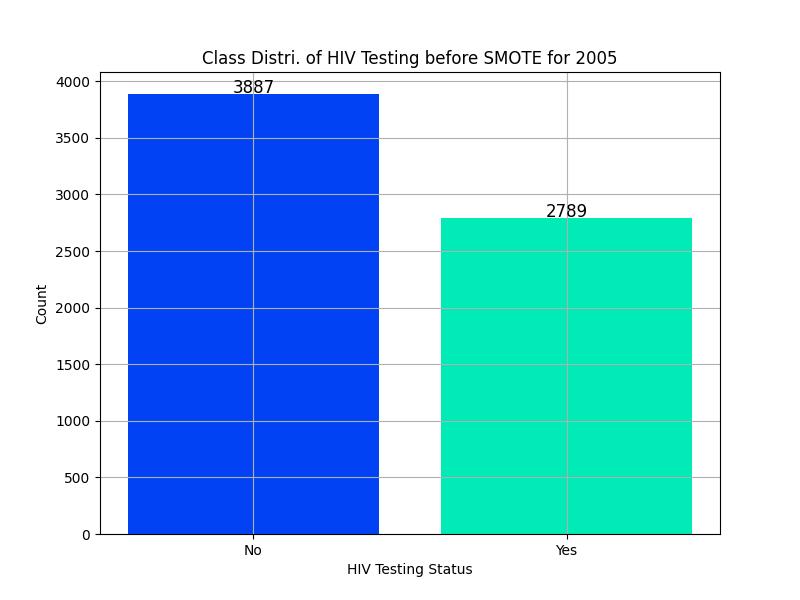

Supplement: Supplementary file 1 [file tropicalmed-10-00167-s001.zip › Figure_S3_Classical_Distribution of HIV Testing_Before_After_SMOTE/balance_before_smote_2005.jpg]

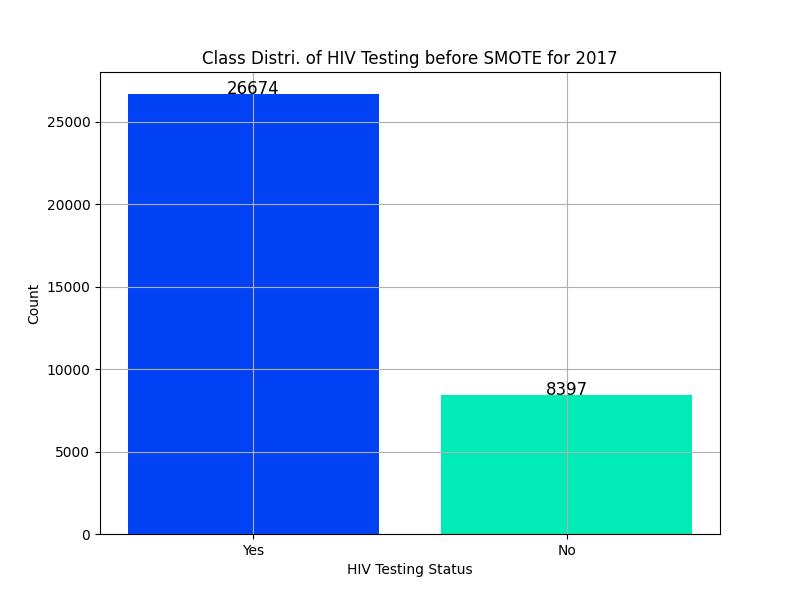

Supplement: Supplementary file 1 [file tropicalmed-10-00167-s001.zip › Figure_S3_Classical_Distribution of HIV Testing_Before_After_SMOTE/balance_before_smote_2017.jpg]
